# The association between partner bereavement and melanoma: cohort studies in the U.K. and Denmark

**DOI:** 10.1111/bjd.18889

**Published:** 2020-03-03

**Authors:** A.Y.S. Wong, T. Frøslev, L. Dearing, H.J. Forbes, A. Mulick, K.E. Mansfield, R.J. Silverwood, A. Kjærsgaard, H.T. Sørensen, L. Smeeth, A. Lewin, S.A.J. Schmidt, S.M. Langan

**Affiliations:** ^1^ Faculty of Epidemiology and Population Health, London School of Hygiene and Tropical Medicine London U.K; ^2^ Department of Clinical Epidemiology Aarhus University Hospital Aarhus Denmark; ^3^ Department of Dermatology Aarhus University Hospital Aarhus Denmark; ^4^ Health Data Research UK London U.K; ^5^ Centre for Longitudinal Studies Department of Social Science University College London London U.K

## Abstract

**Background:**

Psychological stress is commonly cited as a risk factor for melanoma, but clinical evidence is limited.

**Objectives:**

This study aimed to evaluate the association between partner bereavement and (i) first‐time melanoma diagnosis and (ii) mortality in patients with melanoma.

**Methods:**

We conducted two cohort studies using data from the U.K. Clinical Practice Research Datalink (1997–2017) and Danish nationwide registries (1997–2016). In study 1, we compared the risk of first melanoma diagnosis in bereaved vs. matched nonbereaved people using stratified Cox regression. In study 2 we estimated hazard ratios (HRs) for death from melanoma in bereaved compared with nonbereaved individuals with melanoma using Cox regression. We estimated HRs separately for the U.K. and for Denmark, and then pooled the data to perform a random‐effects meta‐analysis.

**Results:**

In study 1, the pooled adjusted HR for the association between partner bereavement and melanoma diagnosis was 0·88 [95% confidence interval (CI) 0·84–0·92] across the entire follow‐up period. In study 2, we observed increased melanoma‐specific mortality in people experiencing partner bereavement across the entire follow‐up period (HR 1·17, 95% CI 1·06–1·30), with the peak occurring during the first year of follow‐up (HR 1·31, 95% CI 1·07–1·60).

**Conclusions:**

We found decreased risk of melanoma diagnosis, but increased mortality associated with partner bereavement. These findings may be partly explained by delayed detection resulting from the loss of a partner who could notice skin changes. Stress may play a role in melanoma progression. Our findings indicate the need for a low threshold for skin examination in individuals whose partners have died.

**What is already known about this topic?**

- Psychological stress has been proposed as a risk factor for the development and progression of cancer, including melanoma, but evidence is conflicting.
- Clinical evidence is limited by small sample sizes, potential recall bias associated with self‐report, and heterogeneous stress definitions.

**What does this study add?**

- We found a decreased risk of melanoma diagnosis, but increased mortality associated with partner bereavement.
- While stress might play a role in the progression of melanoma, an alternative explanation is that bereaved people no longer have a close person to help notice skin changes, leading to delayed melanoma detection.

**Linked Comment:** Talaganis et al. *Br J Dermatol* 2020; **183**:607–608.

## Abstract 2

**Linked Comment:** Talaganis et al. *Br J Dermatol* 2020; **183**:607–608.

Plain language summary available online

Melanoma is a skin cancer characterized by abnormal growth of melanocytes in an existing mole (naevus‐associated melanoma) or on normal skin (*de novo* melanoma). Intense sun exposure, pigmentary traits and family history of skin cancer are known risk factors for melanoma.^1^, ^2^, ^3^ It is estimated that 197 000 new cases of melanoma are diagnosed globally each year, accounting for 1·6% of all incident cancers.^4^ In the U.K. and Denmark, new cases of melanoma account for 5–6% of all cancers, with approximately 16 000 incident cases diagnosed each year in the U.K. and 2330 in Denmark.^5^, ^6^ Early melanoma detection and treatment can improve survival. In Denmark, the 5‐year survival rate with melanoma is 90–94%.^5^ In England, the 5‐year survival rate is 92% in patients with thin tumours (Breslow thickness < 1·5 mm) but only 42% in those with thick tumours (Breslow thickness > 4·0 mm).^7^

Partner bereavement is perceived as one of the most stressful life events.^8^, ^9^, ^10^ Psychological stress has been proposed as a risk factor for the development and progression of cancer, including melanoma, but evidence is conflicting.^11^, ^12^, ^13^, ^14^, ^15^, ^16^ Several physiological pathways have been proposed that implicate stress hormones in carcinogenesis through effects on immune surveillance.^11^, ^13^, ^17^, ^18^, ^19^ However, clinical evidence for such association is limited by small sample sizes, potential recall bias associated with self‐report, and heterogeneous stress definitions.^20^, ^21^, ^22^, ^23^, ^24^, ^25^ Aside from stress, recent studies suggest that having a partner can enhance early detection of melanoma.^26^, ^27^, ^28^ However, we do not know whether loss of a partner negatively affects the incidence and prognosis of melanoma.

We used U.K. and Danish routinely collected data to conduct population‐based cohort studies to evaluate associations between partner bereavement and (i) diagnosis of incident melanoma and (ii) melanoma‐specific mortality. We also investigated whether the associations differed by time since bereavement and whether partner loss was expected.

## Patients and methods

### Settings

Study data were from the U.K. (January 1997 to July 2017) and Denmark (January 1997 to December 2016). Both countries provide universal health coverage from publicly funded healthcare systems.^29^, ^30^

In the U.K., we used Clinical Practice Research Datalink (CPRD) Gold^31^ primary care data with linked mortality (Office for National Statistics, ONS), hospital admission (Hospital Episode Statistics, HES) and deprivation data (Index of Multiple Deprivation) (Appendix S1; see Supporting Information).

We used Danish nationwide registries to obtain data on (i) demographics, civil status and vital status (Civil Registration System),^32^ (ii) incident melanoma (Danish Cancer Registry),^33^ (iii) causes of death (Danish Registry of Causes of Death),^34^ (iv) diagnoses (Danish National Patient Registry),^35^ (v) dispensed prescriptions (Danish National Prescription Registry)^36^ and (vi) education duration (Danish Education Registries).^37^ Data were linked using the unique personal identifier assigned to all Danish residents at birth or immigration. We endeavoured to make the U.K. and Danish studies as similar as possible to ensure comparability (Appendix S1).

### Study 1. Melanoma incidence analysis

We examined the association between partner bereavement and diagnosis of incident melanoma using a matched‐cohort study comparing the risk of melanoma diagnosis in bereaved individuals with that in matched nonbereaved individuals.

In the U.K., we identified eligible couples aged 30 years and over using a previously reported algorithm,^38^, ^39^, ^40^, ^41^, ^42^ while in Denmark we used an algorithm provided by Statistics Denmark (Appendix S2; see Supporting Information). Among eligible couples, we identified a partner as bereaved (exposed) when their partner died, and the bereavement date was the index date. In the U.K., we obtained dates of death from ONS when available (59·8%) and from CPRD for persons not linked to ONS (40·2%). In Denmark, we used death dates from the Civil Registration System. For each bereaved person, we identified a matched comparison cohort who had not previously experienced partner bereavement by sampling (with replacement) up to 10 partners on age (within 1 year) and sex (both settings), county of residence (Denmark) and general practice (U.K.) on the index date. We excluded all individuals who died on the index date as they did not contribute person‐time. We also excluded all individuals with a diagnosis of melanoma before the index date. We required study participants to have ≥ 1 year of healthcare registration history prior to the index date in the U.K., to allow adequate time for recording of covariates and history of melanoma.

The outcome was the first‐ever recorded diagnosis of melanoma (Data Compass for the U.K.^43^ and Appendix S3 for Denmark; see Supporting Information). We followed all cohort members from the index date until the first of: a melanoma diagnosis, date of last data collection from a member's practice (U.K.), transfer out of the practice by either member of the couple (U.K.), emigration of either member of the couple (Denmark), death or the study end date. If a person in the comparison cohort experienced bereavement, he or she was censored 1 day before bereavement and subsequently included in the bereaved cohort (Fig. S1; see Supporting Information).

### Study 2. Melanoma mortality analysis

To assess the association between partner bereavement and melanoma‐specific mortality, we identified a cohort of people diagnosed with melanoma who had partners. We started follow‐up on the date of melanoma diagnosis (Fig. S2; see Supporting Information).

Our main outcome was melanoma‐specific mortality (Appendix S4; see Supporting Information). We included all‐cause mortality as a secondary outcome. In this analysis, we started follow‐up on the date of melanoma diagnosis and ended at the earliest of: the date of last data collection from the patient's practice (U.K.), transfer out of the practice by either member of the couple (U.K.), emigration of either member of the couple (Denmark), death or the study end date.

### Covariates

As possible confounders, we included comorbidities [original Charlson Comorbidity Index (CCI) score],^44^ lifestyle covariates (smoking and alcohol consumption), body mass index and socioeconomic status (Index of Multiple Deprivation status and education duration) (Appendix S5; see Supporting Information). We hypothesized that the level of stress associated with bereavement may depend on whether a partner's death was unexpected. Therefore, we stratified the estimates by the degree to which the partner's death might be considered unexpected based on the level of comorbidity (age‐adjusted CCI score for the deceased partner). As an alternative measure, we identified the presence of terminal disease among partners recorded before the date of death.

### Statistical analysis

We examined descriptive characteristics for different study cohorts on the follow‐up start date. We used Cox regression (with time since cohort entry as the underlying timescale) to estimate hazard ratios (HRs) with 95% confidence intervals (CIs) for the association between partner bereavement and (i) melanoma incidence and (ii) melanoma‐specific mortality. We examined associations for the entire follow‐up period, and by time since start of follow‐up (0–1 year, 0–2 years, 0–3 years, 0–4 years and 0–5 years) to detect any variation due to time lag in the effect of bereavement on the outcome for the *incidence analysis* and to explore the time effect of bereavement since melanoma diagnosis for the *mortality analysis*. For the incidence analysis, we stratified regression models by matched set; thus, unadjusted HRs accounted for matching factors. In sequential models, we estimated HRs adjusted for participants’ CCI level (adjusted model) and then added lifestyle variables and deprivation status (U.K.) and education duration (Denmark) (fully adjusted model).

We assessed the assumption of proportional hazards by visual inspection of log–log plots (Fig. S3; see Supporting Information). Additionally, we examined HRs over time by stratifying the follow‐up period since bereavement (0–1 year, 1–2 years, 2–3 years, 3–4 years, 4–5 years, ≥ 5 years) (Table S1; see Supporting Information).

We also examined variation by age at index date, sex and risk of partner death (deceased partner's age‐adjusted CCI score and terminal disease) and performed likelihood ratio tests to explore possible effect modification by these characteristics.

For the mortality analysis, we included time‐varying bereavement as the exposure in the unadjusted model. In the adjusted model, we also adjusted for age, sex and CCI score; and in the fully adjusted model we additionally adjusted for lifestyle and socioeconomic variables. We also examined the association between bereavement and melanoma‐specific mortality in categories of cancer stage at diagnosis (localized, regional, distant) among patients with this information recorded in the Danish Cancer Registry. Finally, we assessed the association between bereavement and mortality according to age at melanoma diagnosis and sex, and performed likelihood ratio tests to analyse effect modification.

In both analyses, we undertook complete‐case analyses in the fully adjusted models, which would be unbiased assuming that missingness was not associated with the outcome conditional on the other variables. As lifestyle data (used in U.K. analyses only) are unlikely to be missing at random and we lacked data on probable predictors of missingness, imputation techniques were not appropriate for correcting potential biases.^45^ For the incidence analysis, we further investigated patterns of missing data using conditional logistic regression. We conducted several sensitivity analyses to test the robustness of the results in both the incidence and mortality analyses (Table S2; see Supporting Information). All study analyses were preplanned unless otherwise stated.

We conducted all analyses separately for the U.K. (using Stata/MP 15·1; StataCorp, College Station, TX, U.S.A.) and Denmark (using SAS 9·4; SAS Institute Inc., Cary, NC, U.S.A.). We combined the main results (from the adjusted models) in Stata using the DerSimonian and Lairds’ random‐effects model.^46^

## Results

### Study 1. Melanoma incidence analysis

**Figure 1:**
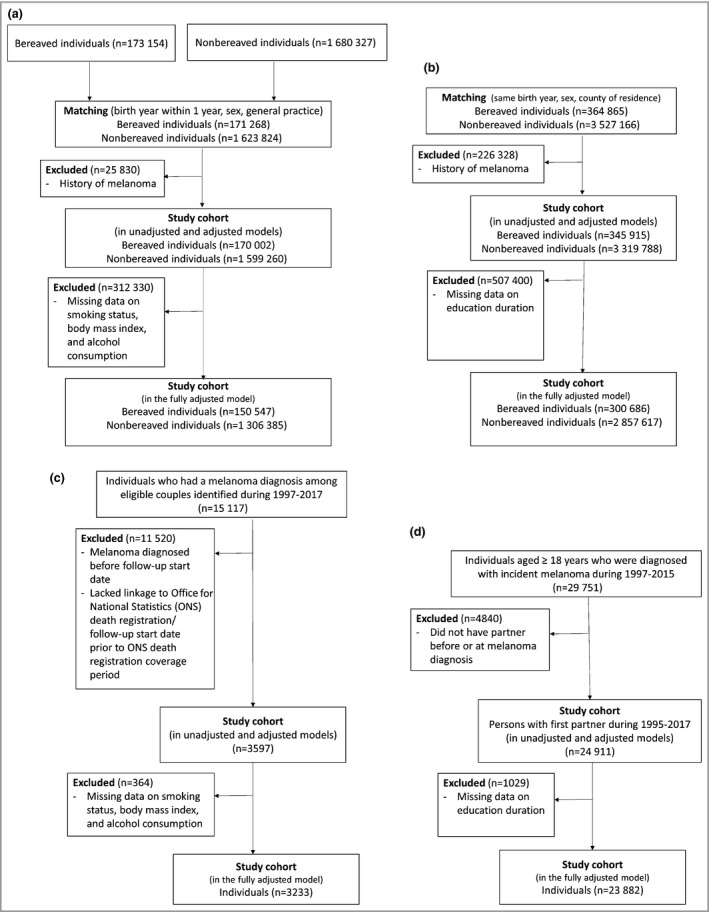
Flowcharts for inclusion in the cohorts in the U.K. and Denmark. (a) Incidence analysis in the U.K., (b) incidence analysis in Denmark, (c) mortality analysis in the U.K., (d) mortality analysis in Denmark.

Study 1 included 170 002 bereaved and 1 599 260 matched nonbereaved individuals in the U.K., and 345 915 bereaved and 3 319 788 matched nonbereaved individuals in Denmark (Fig. 1). The median age was 74 years in the U.K. and 71 years in Denmark. Approximately two‐thirds of both cohorts were women (Table 1). Bereaved people were more likely to have higher CCI scores, to be more deprived, to have a shorter education, and to have slightly longer median follow‐up than people in the comparison cohort.

**Table 1:** Study 1: characteristics of the bereaved and matched comparison cohorts used in the melanoma incidence analysis

	U.K.		Denmark	
	Bereaved cohort	Comparison cohort^a^	Bereaved cohort	Comparison cohort^a^
Total	170 002 (9·6)	1 599 260 (90·4)	345 915 (9·4)	3 319 788 (90·6)
Age at index date (years)				
Range	31·9–101·4	31·4–100·4	16·5–100·0	16·1–99·9
Median (IQR)	74·5 (66·8–80·8)	73·8 (66·3–79·8)	71·3 (62·4–78·8)	70·8 (62·0–78·0)
Groups				
< 50	3081 (1·8)	30 096 (1·9)	23 956 (6·9)	238 640 (7·2)
50–59	15 843 (9·3)	158 537 (9·9)	45 143 (13·1)	449 727 (13·5)
60–69	39 239 (23·1)	391 003 (24·5)	89 214 (25·8)	887 777 (26·7)
70–79	64 000 (37·7)	630 668 (39·4)	114 708 (33·2)	1 123 948 (33·9)
≥ 80	47 839 (28·1)	388 956 (24·3)	72 894 (21·1)	619 696 (18·7)
Sex female				
Female	111 427 (65·5)	1 048 995 (65·6)	231 022 (66·8)	2 214 531 (66·7)
Male	58 575 (34·5)	550 265 (34·4)	114 893 (33·2)	1 105 257 (33·3)
Comorbidity burden^b^				
Low	78 347 (46·1)	773 297 (48·4)	249 026 (72·0)	2 458 135 (74·0)
Intermediate	62 126 (36·5)	571 089 (35·7)	81 430 (23·5)	728 846 (22·0)
High	29 529 (17·4)	254 874 (15·9)	15 459 (4·5)	132 807 (4·0)
Smoking status^c^				
Never smoked	61 330 (36·1)	624 987 (39·1)	NA	NA
Formerly smoked	69 069 (40·6)	666 389 (41·7)	NA	NA
Currently smokes	36 862 (21·7)	286 561 (17·9)	NA	NA
Missing	2741 (1·6)	21 323 (1·3)	NA	NA
Alcohol consumption^c^				
Never drank	19 913 (11·7)	169 930 (10·6)	NA	NA
Formerly drank	22 128 (13·0)	185 976 (11·6)	NA	NA
Currently drinks	114 823 (67·5)	1 134 558 (70·9)	NA	NA
Missing	13 138 (7·7)	108 796 (6·8)	NA	NA
Body mass index (kg m^−2^)^c^				
< 18·5	4216 (2·5)	28 321 (1·8)	NA	NA
18·5–24·9	57 830 (34·0)	544 495 (34·1)	NA	NA
25–29·9	58 967 (34·7)	590 334 (36·9)	NA	NA
≥ 30	35 856 (21·1)	333 589 (20·9)	NA	NA
Missing	13 133 (7·7)	102 521 (6·4)	NA	NA
Index of multiple deprivation^c^				
1 (least deprived)	39 713 (23·4)	400 092 (25·0)	NA	NA
2	35 361 (20·8)	345 884 (21·6)	NA	NA
3	36 653 (21·6)	344 956 (21·6)	NA	NA
4	33 049 (19·4)	292 864 (18·3)	NA	NA
5 (most deprived)	25 226 (14·8)	215 464 (13·5)	NA	NA
Education duration (years)^d^				
Short (7–10)	NA	NA	157 611 (45·6)	1 370 756 (41·3)
Medium (11–12)	NA	NA	103 144 (29·8)	1 058 069 (31·9)
Long (≥ 13)	NA	NA	40 506 (11·7)	526 196 (15·9)
Missing	NA	NA	44 654 (12·9)	364 767 (11·0)
Follow‐up (years)				
Total	905 281	8 137 952	2 552 711	22 027 622
Median (IQR)	4·3 (1·8–8·1)	4·1 (1·8–7·5)	6·6 (3·0–11·2)	5·6 (2·5–10·0)

The data are presented as *n* (%) unless stated otherwise. IQR, interquartile range; NA, not applicable. ^a^In the U.K. comparison cohort, 18·7% (15·1% of unique individuals) experienced bereavement after the end of follow‐up. In the Danish comparison cohort, 22·7% (17·0% of unique individuals) experienced bereavement after the end of follow‐up. ^b^Comorbidity burden was measured using the Charlson Comorbidity Index. Comorbidity burden was determined on the index date based on the Charlson Comorbidity Index score, categorized as low (0 point), intermediate (1–2 points) or high (≥ 3 points). ^c^Information on smoking status, alcohol consumption, body mass index and Index of Multiple Deprivation was not available in Denmark. ^d^Information on education duration was not available in the U.K.

**Figure 2:**
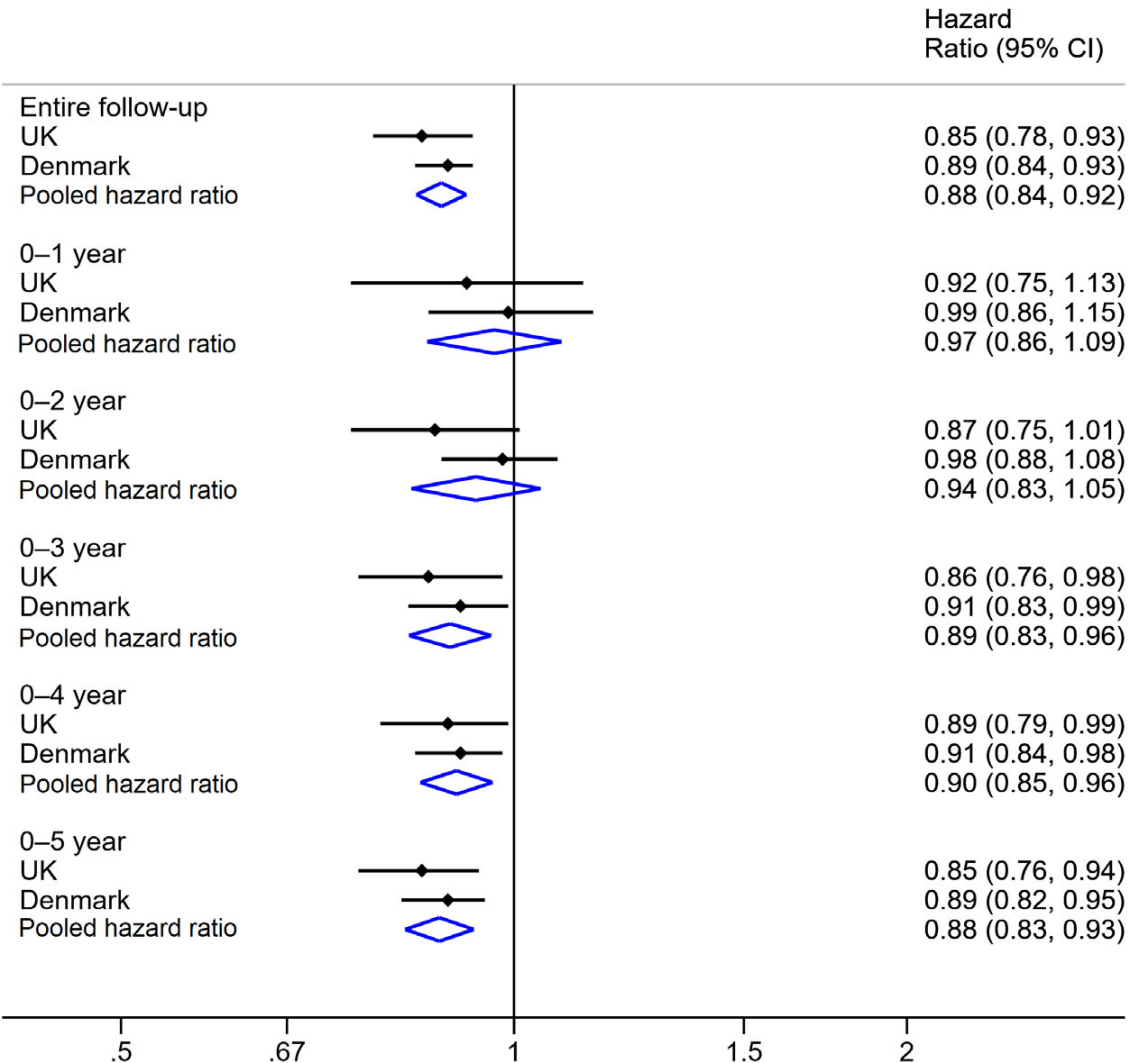
Pooled adjusted hazard ratios and confidence intervals (CIs) for the association between partner bereavement and diagnosis of incident melanoma in the U.K. and Denmark. Hazard ratios were adjusted for Charlson Comorbidity Index scores.

The pooled HR (adjusted for study participants’ CCI scores) comparing melanoma diagnosis rates in bereaved vs. nonbereaved individuals was 0·88 (95% CI 0·84–0·92) (Fig. 2). We did not find evidence of lower HRs for melanoma within 0–1 year (HR 0·97, 95% CI 0·86–1·09) or 0–2 years (HR 0·94, 95% CI 0·83–1·05). However, we found evidence of a lower melanoma rate following partner bereavement within 0–3 years (HR 0·89, 95% CI 0·83–0·96), 0–4 years (HR 0·90, 95% CI 0·85–0·96) and 0–5 years (HR 0·88, 95% CI 0·83–0·93) of follow‐up. Estimates were similar in the fully adjusted models (Table S3; see Supporting Information).

We found evidence of effect modification by age in the U.K. but not in Denmark (Table S4; see Supporting Information). We observed no substantial variation by sex or whether the partner's death was foreseen, in either country.

In the U.K., missing lifestyle data were dependent on incident melanoma, conditional on bereavement status and other covariates (Table S5; see Supporting Information). However, HRs for the whole cohort and the complete‐case cohort were similar in the unadjusted and adjusted models in both countries (Table S6; see Supporting Information). The results of sensitivity analyses were broadly similar to those of the main analyses (Tables S7–12; see Supporting Information).

### Study 2. Melanoma mortality analysis

We followed 3597 patients with melanoma in the U.K. and 24 911 people with melanoma in Denmark (Fig. 1). The median follow‐up time was 3·5 years in the U.K. and 5·0 years in Denmark (Table 2). More people who were aged < 50 years and had fewer comorbidities were included in Denmark compared with the U.K. In Denmark, most individuals had localized cancer at diagnosis (74·6%). Among 2162 individuals who experienced bereavement on or prior to melanoma diagnosis, 1485 (68·7%) had localized melanoma, 135 (6·2%) had regional melanoma and 24 (1·1%) had distant cancer at diagnosis.

**Table 2:** Study 2: characteristics of patients with melanoma among couples in the mortality analysis

	U.K.	Denmark
Total	3597	24 911
Age (years)		
Range	32·8–99·0	18·3–99·5
Median (IQR)	67·2 (58·2–75·5)	58·7 (45·3–69·8)
Groups		
< 50	283 (7·9)	8276 (33·2)
50–59	782 (21·7)	4888 (19·6)
60–69	1092 (30·4)	5633 (22·6)
70–79	958 (26·6)	4051 (16·3)
≥ 80	482 (13·4)	2063 (8·3)
Sex		
Female	1606 (44·7)	13 035 (52·3)
Male	1991 (55·4)	11 876 (47·7)
Comorbidity burden^a^		
Low	1858 (51·7)	20 254 (81·3)
Intermediate	1117 (31·1)	3847 (15·4)
High	622 (17·3)	810 (3·3)
Smoking status^b^		
Never smoked	1415 (39·3)	NA
Formerly smoked	1559 (43·3)	NA
Currently smokes	595 (16·5)	NA
Missing	28 (0·8)	NA
Alcohol consumption^b^		
Never drank	236 (6·6)	NA
Formerly drank	266 (7·4)	NA
Currently drinks	2832 (78·7)	NA
Never drank	263 (7·3)	NA
Body mass index (kg m^−2^)^b^		
< 18·5	43 (1·2)	NA
18·5–24·9	1172 (32·6)	NA
25–29·9	1405 (39·1)	NA
≥ 30	754 (21·0)	NA
Missing	223 (6·2)	NA
Index of Multiple Deprivation^b^		
1 (least deprived)	1099 (30·6)	NA
2	1019 (28·3)	NA
3	788 (21·9)	NA
4	522 (14·5)	NA
5 (most deprived)	169 (4·7)	NA
Education duration (years)^c^		
Short (7–10)	NA	5909 (23·7)
Medium (11–12)	NA	10 410 (41·8)
Long (≥ 13)	NA	7563 (30·4)
Missing	NA	1029 (4·1)
Melanoma stage at diagnosis^c^		
Localized	NA	18 575 (74·6)
Regional	NA	1500 (6·0)
Distant	NA	254 (1·0)
Unknown	NA	4582 (18·4)
Follow‐up (years)		
Total	17 625	154 189
Median (IQR)	3·5 (1·4–6·8)	5·0 (2·2–9·3)

The data are presented as *n* (%) unless stated otherwise. IQR, interquartile range; NA, not applicable. ^a^Comorbidity burden was measured using the Charlson Comorbidity Index. Comorbidity burden was determined on the index date using the Charlson Comorbidity Index score, categorized as low (0 point), intermediate (1–2 points) or high (≥ 3 points). ^b^Information on smoking status, alcohol consumption, body mass index and Index of Multiple Deprivation was not available in Denmark. ^c^Information on education duration and melanoma stage at diagnosis was not available in the U.K.

**Figure 3:**
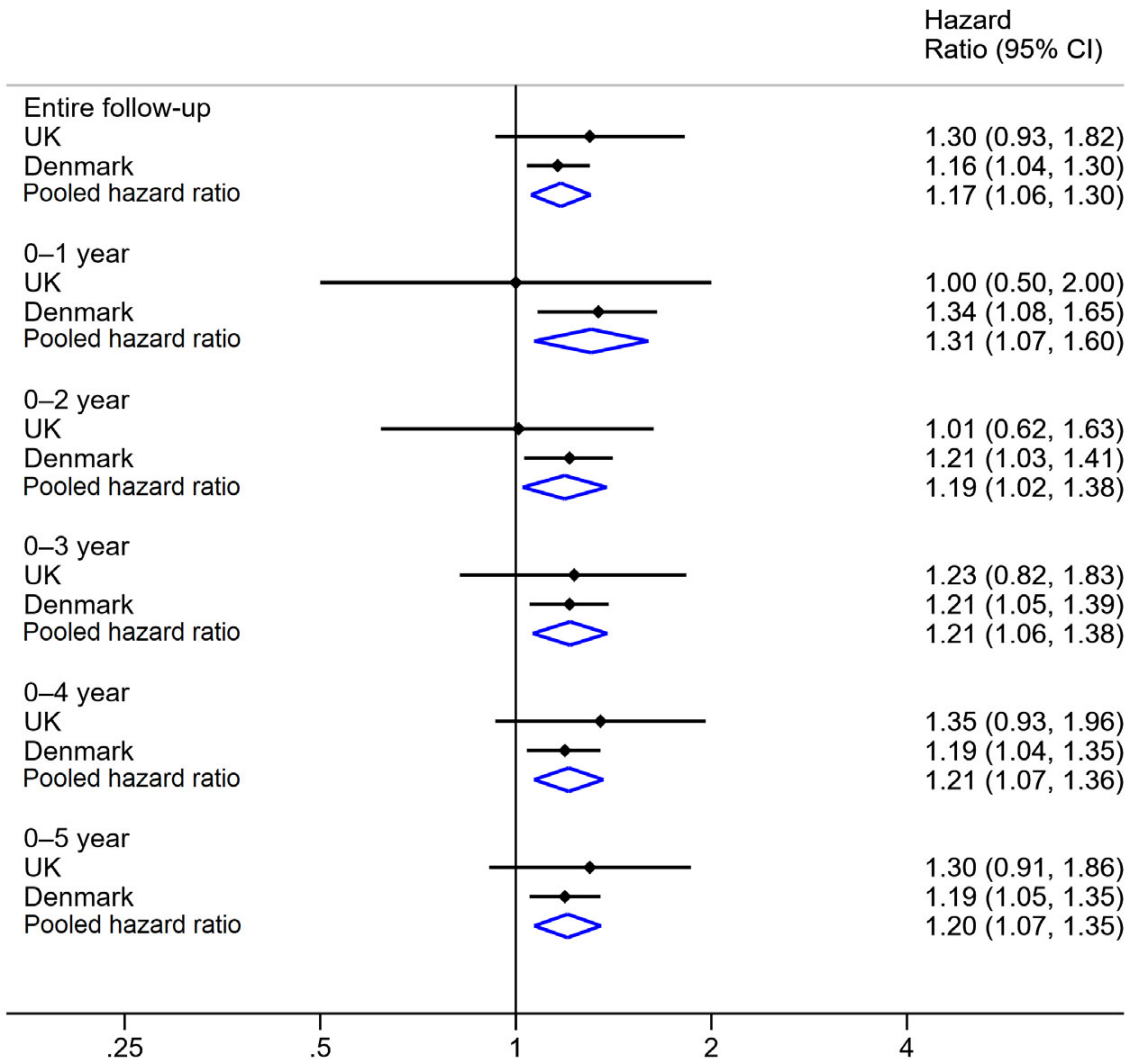
Pooled adjusted hazard ratios and confidence intervals (CIs) for the association between partner bereavement and melanoma‐specific mortality among patients with melanoma in the U.K. and Denmark. Hazard ratios were adjusted for age, sex and Charlson Comorbidity Index scores.

After adjusting for age, sex and study participants’ CCI score, we observed increased melanoma‐specific mortality in those with partner bereavement (pooled HR 1·17, 95% CI 1·06–1·30) compared with those without (Fig. 3). The analysis by time since melanoma diagnosis showed that the increased HR for melanoma‐specific mortality in the bereaved vs. nonbereaved cohorts peaked within 0–1 year (HR 1·31 95% CI 1·07–1·60) of follow‐up and remained stable during 0–2 years (HR 1·19, 95% CI 1·02–1·38), 0–3 years (HR 1·21, 95% CI 1·06–1·38), 0–4 years (HR 1·21, 95% CI 1·07–1·36) and 0–5 years (HR 1·20, 95% CI 1·07–1·35) of follow‐up. Similar HRs were observed in the fully adjusted models (Table S13; see Supporting Information). HRs generated by unadjusted and adjusted models for the whole cohort and the complete‐case cohort were similar in both countries (Table S14; see Supporting Information). Additionally, we observed approximately a 20–30% increased hazard of all‐cause mortality associated with partner bereavement during the entire follow‐up period in both countries (Table S15; see Supporting Information).

Wide CIs were observed for all subgroups due to small sample sizes (Table S16; see Supporting Information). In Denmark, we did not find evidence of effect modification by cancer stage (Table S17; see Supporting Information). The results of all other sensitivity analyses were similar to those in the main analysis (Tables S18–24; see Supporting Information).

## Discussion

This study showed that partner bereavement was associated with a 12% decreased risk of being diagnosed with incident melanoma in two large population‐based studies. We observed an increase in melanoma‐specific mortality associated with partner bereavement, which peaked during the first year following melanoma diagnosis.

Several studies have examined the role of other stressors in melanoma incidence, but no studies have focused on partner bereavement and melanoma.^12^, ^20^, ^21^ A meta‐analysis showed no association between risk of skin cancers, including melanoma, and stress‐related psychosocial factors such as stressful life events, severe chronic stress and daily stress.^12^ However, the review did not assess studies focusing on melanoma only. In contrast, a case–control study assessing self‐reported loss of a relative or friend in the past year reported an increased risk of melanoma in bereaved individuals.^20^ Our observed lower rate of melanoma diagnosis in bereaved people may reflect delayed melanoma detection after partner loss.

Supporting this theory, a recent randomized controlled trial reported that providing a structured skin self‐examination education intervention to patients with prior melanoma and their partners resulted in identification of more melanomas compared with customary care, including identification of more *in situ* melanomas.^26^ Another study reported that people married at melanoma diagnosis were two to three times more likely to have a thinner tumour than nonmarried individuals.^28^ A cohort study based on data from the U.S. National Cancer Institute's Surveillance, Epidemiology, and End Results database also showed that widowed people were less likely to undergo sentinel lymph node biopsy and were more likely to present with a higher stage of melanoma compared with married people.^27^ These studies suggest that partner loss could decrease early diagnosis of melanoma, which is consistent with our findings. Social isolation, residual socioeconomic confounding, reduced self‐care and reduced likelihood of seeking medical attention following bereavement may also have contributed to the lower incidence of diagnosed melanoma we observed. Our study highlights the importance of encouraging family members or caregivers to perform skin examinations for bereaved persons.

It has been suggested that stress hormones can accelerate growth and migration of tumour cells, worsening melanoma prognosis, as immunological surveillance is important in melanoma outcomes.^13^, ^17^, ^18^ Consistently with our findings, two small studies reported that a range of positive psychosocial factors (including marriage) predicted longer survival following melanoma,^22^, ^25^ while another found no association with time to relapse among 155 patients with melanoma or breast cancer.^23^, ^24^ A meta‐analysis showed no significant effects of stress‐related psychosocial factors on skin cancer survival (melanoma and nonmelanoma).^12^ All of these prior studies had limitations including inadequate power, inclusion of a wide range of psychological constructs, and lack of control for other risk factors,^22^, ^23^, ^24^, ^25^, ^47^ but the results were similar to those in our study. A previous study^38^ reported a short‐term increased risk of cardiovascular events within 90 days after partner bereavement, suggesting that cardiovascular events may partly explain our observation of increased all‐cause mortality up to 5 years following bereavement, although some of these deaths may represent misclassified melanoma‐specific mortality.

Apart from stress, delayed detection of recurrence or a secondary melanoma due to lack of an available partner to notice skin changes might also account for our findings. Unfortunately, our stage‐specific analyses in Denmark were associated with large statistical imprecision, precluding firm conclusions. Previous studies have shown that those without a partner experienced higher death rates,^48^ shorter survival^49^, ^50^, ^51^, ^52^, ^53^ and more advanced stage of melanoma at time of diagnosis.^51^, ^54^, ^55^ However, most studies have focused on women only^48^, ^49^ or lacked adjustment for lifestyle factors^52^, ^53^ or socioeconomic status.^50^, ^51^

Combining population‐based data from two countries (U.K. and Denmark) provides credibility to our findings by demonstrating replicability, attaining a greater sample size, exploring various sources of bias (e.g. confounding by lifestyle factors) and using validated outcomes. Validation studies have shown high positive predictive values (≥ 83%) of identifying cases of melanoma based on data in both the CPRD and the Danish Cancer Registry.^56^, ^57^

To control for potential confounding, we adjusted our analyses for socioeconomic status and lifestyle variables. However, we did not have information on some risk factors for melanoma including sun exposure, pigmentary traits and family history of skin cancer. Residual confounding is a possibility. We matched our cohort with replacement in the main analysis in both settings, which might have led to narrower CIs. Excluding people with missing lifestyle information in the U.K. had minimal effects on estimates, implying that these missing data were unlikely to have affected our interpretation of the results. Misclassification of partnership also could have occurred, including changes in partner status over time. Particularly in the U.K., where direct data on partnership status were not available, this may have led to nondifferential misclassification and underestimation of any association. However, we used relatively strict criteria (e.g. age difference of members of the couples) to identify partners in the U.K., to minimize such misclassification.^38^, ^39^, ^40^, ^41^ Importantly, longitudinal data on partnership were available in the Danish study, and the findings were broadly similar to those of the U.K. study.

In conclusion, we observed a lower risk of a melanoma diagnosis following partner bereavement. This finding might be explained by delayed detection in the absence of a partner's help with skin examinations among the bereaved. This mechanism could also explain the increase in melanoma mortality associated with partner bereavement, although stress might promote melanoma progression. Our findings highlight the need to raise public awareness of the association to promote self‐skin examination, and to encourage clinicians to have a lower threshold for undertaking skin examinations in bereaved people.

## Supporting information


**Appendix S1** Detailed information on data sources.
**Appendix S2** Partner algorithms.
**Appendix S3** Code lists used to define partners’ risk of death, outcomes and other covariates in Denmark.
**Appendix S4** Identification of melanoma mortality.
**Appendix S5** Details on covariates.
**Fig S1.** Illustration of follow‐up in the melanoma incidence analysis.
**Fig S2.** Illustration of follow‐up in the melanoma mortality analysis.
**Fig S3.** Assessment of the assumption of proportional hazards.
**Table S1** Results of stratifying follow‐up time since partner bereavement in the melanoma incidence analysis.
**Table S2** List of sensitivity analyses.
**Table S3** Association between partner bereavement and diagnosis of incident melanoma, overall and by time since the follow‐up start date.
**Table S4** Association between partner bereavement and diagnosis of incident melanoma, subgroup analysis.
**Table S5** Patterns of missingness of smoking status, body mass index and alcohol consumption data in the melanoma incidence analysis.
**Table S6** Association between partner bereavement and diagnosis of incident melanoma. Unadjusted and adjusted hazard ratios for the full cohort and the complete‐case cohort.
**Table S7** Association between partner bereavement and incident melanoma, sensitivity analysis restricted to patients with more than 5 years of registration history prior to the index date.
**Table S8** Association between partner bereavement and diagnosis of incident melanoma, sensitivity analysis restricted to patients eligible for linkage to Hospital Episode Statistics or Office for National Statistics death registration data.
**Table S9** Association between partner bereavement and incident melanoma, post hoc intention‐to‐treat analysis.
**Table S10** Association between partner bereavement and diagnosis of incident melanoma, post hoc sensitivity analysis redefining the cohort using matching without replacement in the U.K.
**Table S11** Association between partner bereavement and diagnosis of incident melanoma, sensitivity analysis censoring at the end of partnership.
**Table S12** Association between partner bereavement and diagnosis of incident melanoma, sensitivity analysis including only histologically verified diagnoses in the outcome definition.
**Table S13** Association between partner bereavement and melanoma mortality in patients with melanoma, overall and by time since melanoma diagnosis.
**Table S14** Association between partner bereavement and mortality in patients with melanoma. Unadjusted and adjusted hazard ratios for the full cohort and the complete‐case cohort.
**Table S15** Association between partner bereavement and all‐cause mortality in patients with melanoma, overall and by time since melanoma diagnosis, post hoc.
**Table S16** Association between partner bereavement and melanoma mortality in patients with melanoma, subgroup analysis by age and sex.
**Table S17** Association between partner bereavement and melanoma‐specific mortality in patients with melanoma, subgroup analysis by cancer stage at diagnosis in Denmark.
**Table S18** Characteristics of patients with melanoma in the U.K. (not limited to those with data linkage to the Office of National Statistics death registration).
**Table S19** Association between partner bereavement and all‐cause mortality among patients with melanoma (not limited to those with data linkage to the Office for National Statistics death registration), post hoc.
**Table S20** Association between partner bereavement and melanoma mortality in patients with melanoma, sensitivity analysis excluding those who experienced bereavement before or on the date of melanoma diagnosis.
**Table S21** Association between partner bereavement and melanoma mortality, sensitivity analysis post hoc excluding patients who had lost their partner or were no longer in a partnership with their partner 3 years prior to melanoma diagnosis.
**Table S22** Association between partner bereavement and melanoma mortality, sensitivity analysis censoring follow‐up at the end of the partnership, and excluding persons if this occurred before melanoma diagnosis.
**Table S23** Association between partner bereavement and melanoma mortality, sensitivity analysis post hoc censoring follow‐up at emigration or end of the partnership, and excluding persons if either event occurred before melanoma diagnosis.
**Table S24** Association between partner bereavement and melanoma mortality, sensitivity analysis including only histologically verified diagnoses in the outcome definition.
