# Supplementary material for: The association between partner bereavement and melanoma: cohort studies in the U.K. and Denmark
Source: Br J Dermatol. 2020 Mar 3;183(4):673–83. doi: 10.1111/bjd.18889 (PMC7587014; doi:10.1111/bjd.18889)
Supplement: Supplementary file 1 — Appendix S1 Detailed information on data sources. Appendix S2 Partner algorithms. Appendix S3 Code lists used to define partners’ risk of death, outcomes and other covariates in Denmark. Appendix S4 Identification of melanoma mortality. Appendix S5 Details on covariates. Fig S1. Illustration of follow‐up in the melanoma incidence analysis. Fig S2. Illustration of follow‐up in the melanoma mortality analysis. Fig S3. Assessment of the assumption of proportional hazards. Table S1 Results of stratifying follow‐up time since partner bereavement in the melanoma incidence analysis. Table S2 List of sensitivity analyses. Table S3 Association between partner bereavement and diagnosis of incident melanoma, overall and by time since the follow‐up start date. Table S4 Association between partner bereavement and diagnosis of incident melanoma, subgroup analysis. Table S5 Patterns of missingness of smoking status, body mass index and alcohol consumption data in the melanoma incidence analysis. Table S6 Association between partner bereavement and diagnosis of incident melanoma. Unadjusted and adjusted hazard ratios for the full cohort and the complete‐case cohort. Table S7 Association between partner bereavement and incident melanoma, sensitivity analysis restricted to patients with more than 5 years of registration history prior to the index date. Table S8 Association between partner bereavement and diagnosis of incident melanoma, sensitivity analysis restricted to patients eligible for linkage to Hospital Episode Statistics or Office for National Statistics death registration data. Table S9 Association between partner bereavement and incident melanoma, post hoc intention‐to‐treat analysis. Table S10 Association between partner bereavement and diagnosis of incident melanoma, post hoc sensitivity analysis redefining the cohort using matching without replacement in the U.K. Table S11 Association between partner bereavement and diagnosis of incident melanoma, sensitivity analysis c [file BJD-183-673-s001.docx]

**Supplementary material: The association between partner bereavement and melanoma: cohort studies in the UK and Denmark**

Contents

[**Supplementary material method 1. Detailed information on data sources.** 4](#_Toc26286744)

[**Supplementary material method 2. Partner algorithms.** 8](#_Toc26286745)

[**Supplementary material method 3. Code lists used to define partners’ risk of death, outcomes, and other covariates in Denmark.** 10](#_Toc26286746)

[**Supplementary material method 4. Identification of melanoma mortality.** 13](#_Toc26286747)

[**Supplementary material method 5. Details on covariates.** 14](#_Toc26286748)

[**Supplementary material figure 1. Illustration of follow-up in the melanoma incidence analysis.** 15](#_Toc26286749)

[**Supplementary material figure 2. Illustration of follow-up in the melanoma mortality analysis.** 16](#_Toc26286750)

[**Supplementary material figure 3. Assessment of the assumption of proportional hazards.** 17](#_Toc26286751)

[**Supplementary material table 1. Results of stratifying follow-up time since partner bereavement in the melanoma incidence analysis.** 19](#_Toc26286752)

[**Supplementary material table 2. List of sensitivity analyses.** 20](#_Toc26286753)

[**Supplementary material table 3. Association between partner bereavement and diagnosis of incident melanoma, overall & by time since the follow-up start date.** 22](#_Toc26286754)

[**Supplementary material table 4. Association between partner bereavement and diagnosis of incident melanoma, subgroup analysis.** 23](#_Toc26286755)

[**Supplementary material table 5. Patterns of missingness of smoking status, body mass index, and alcohol consumption data in the melanoma incidence analysis.** 25](#_Toc26286756)

[**Supplementary material table 6. Association between partner bereavement and diagnosis of incident melanoma. Unadjusted and adjusted hazard ratios for the full cohort and the complete case cohort.** 28](#_Toc26286757)

[**Supplementary material table 7. Association between partner bereavement and incident melanoma, sensitivity analysis restricted to patients with more than 5 years of registration history prior to the index date.** 29](#_Toc26286758)

[**Supplementary material table 8. Association between partner bereavement and diagnosis of incident melanoma, sensitivity analysis restricted to patients eligible for linkage to Hospital Episode Statistics/Office for National Statistics death registration data.** 30](#_Toc26286759)

[**Supplementary material table 9. Association between partner bereavement and incident melanoma, *post-hoc* intention-to-treat analysis.** 31](#_Toc26286760)

[**Supplementary material table 10. Association between partner bereavement and diagnosis of incident melanoma, *post-hoc* sensitivity analysis redefining the cohort using matching without replacement in the UK.** 33](#_Toc26286761)

[**Supplementary material table 11. Association between partner bereavement and diagnosis of incident melanoma, sensitivity analysis censoring at end of partnership.** 34](#_Toc26286762)

[**Supplementary material table 12. Association between partner bereavement and diagnosis of incident melanoma, sensitivity analysis including only histologically verified diagnoses in the outcome definition.** 35](#_Toc26286763)

[**Supplementary material table 13. Association between partner bereavement and melanoma mortality in patients with melanoma, overall and by time since melanoma diagnosis.** 36](#_Toc26286764)

[**Supplementary material table 14. Association between partner bereavement and mortality in patients with melanoma. Unadjusted and adjusted hazard ratios for the full cohort and the complete case cohort.** 37](#_Toc26286765)

[**Supplementary material table 15. Association between partner bereavement and all-cause mortality in patients with melanoma, overall and by time since melanoma diagnosis, *post hoc.*** 38](#_Toc26286766)

[**Supplementary material table 16. Association between partner bereavement and melanoma mortality in patients with melanoma, subgroup analysis by age and sex.** 39](#_Toc26286767)

[**Supplementary material table 17. Association between partner bereavement and melanoma-specific mortality in patients with melanoma, subgroup analysis by cancer stage at diagnosis in Denmark.** 40](#_Toc26286768)

[**Supplementary material table 18. Characteristics of patients with melanoma in the UK (not limited to those with data linkage to the Office of National Statistics death registration).** 41](#_Toc26286769)

[**Supplementary material table 19. Association between partner bereavement and all-cause mortality among patients with melanoma (not limited to those with data linkage to the Office for National Statistics death registration), *post hoc.*** 42](#_Toc26286770)

[**Supplementary material table 20. Association between partner bereavement and melanoma mortality in melanoma patients, sensitivity analysis excluding those who experienced bereavement before or on the date of melanoma diagnosis.** 43](#_Toc26286771)

[**Supplementary material table 21. Association between partner bereavement and melanoma mortality, sensitivity analysis *post hoc* excluding patients who had lost their partner or were no longer in a partnership with their partner 3 years prior to melanoma diagnosis.** 44](#_Toc26286772)

[**Supplementary material table 22. Association between partner bereavement and melanoma mortality, sensitivity analysis censoring follow-up at the end of the partnership, and excluding persons if this occurred before melanoma diagnosis.** 45](#_Toc26286773)

[**Supplementary material table 23. Association between partner bereavement and melanoma mortality, sensitivity analysis *post hoc* censoring follow-up at emigration or end of the partnership, and excluding persons if either event occurred before melanoma diagnosis.** 46](#_Toc26286774)

[**Supplementary material table 24. Association between partner bereavement and melanoma mortality, sensitivity analysis including only histologically verified diagnoses in the outcome definition.** 47](#_Toc26286775)

# **Supplementary material method 1. Detailed information on data sources.**

In the United Kingdom (UK) and Denmark, general practitioners coordinate all healthcare contacts, including referral to specialists.

***UK***

We based the UK cohort study on data from the Clinical Practice Research Datalink (CPRD), Hospital Episode Statistics (HES), the Office for National Statistics (ONS), and the Index of Multiple Deprivation (IMD).

The CPRD covers approximately 7% of the UK population, a representative sample with regards to demographics (age, sex, and ethnicity) [1]. We used data from the CPRD Gold July 2017 build which contains 14,942,430 patients who met acceptable quality standards across 718 general practices. The CPRD contains prospectively collected primary care records recorded from general practices documented using Vision Software. Anonymised data include information on symptoms and diagnoses (coded using Read codes), written prescriptions (coded using British National Formulary codes), health-related behaviors (smoking and alcohol consumption), anthropometric data (height and weight), and referrals to specialists. Approximately 60% of participating practices registered in England have consented to link their patient records to other data sources, including the HES, ONS and IMD.

The HES contains National Health Service inpatient hospital stay records in England, with linkage to the CPRD since 1997. Diagnoses are coded using the International Classification of Diseases, *Tenth Revision* (ICD-10), while procedures, including operations, are coded using the Office of Population and Censuses and Surveys (OPCS) Classification of Interventions and Procedures codes, Version 4 [2]. Several diagnoses and procedures can be recorded for each care episode. Data on the order of diagnosis codes for a given episode are available and the first-listed diagnosis is typically the main reason for admission. Data from hospital outpatient contacts were not available. In this study, we did not restrict the cohort to only those eligible for HES in the main analysis, because the melanoma is commonly diagnosed at the general practice. We tested the robustness of the results by restricting the cohort to those with linked HES only as a sensitivity analysis.

The ONS contains death registration data. Most death registration information is supplied by the informant (usually a close relative of the deceased) while the cause of death is usually obtained from the Medical Certificate of Cause of Death completed by a medical practitioner when the death is certified. Deaths should be registered within five days of the date of death and on average 78% of deaths are registered within this time frame. The death registration data used in our study included all deaths registered from 2 January 1998 to 19 September 2017. Data on dates of death and cause of death (including underlying cause and up to 15 other recorded causes of death) are available. Causes of death have been coded using ICD-9 codes before January 2001 and ICD-10 codes thereafter.

The English Indices of Deprivation [3] are based on a number of indicators covering different aspects of material deprivation (housing, employment, income, access to services, education and skills, crime, living environment). The Index of Multiple Deprivation (IMD) is calculated as a weighted sum of the domain indices. For practices in England that have consented to participate in the linkage scheme, the patient postcode of residence is mapped to the 2001 “lower layer super output area” (LSOA) boundaries using a postcode lookup file. A practice-level linkage uses the practice postcode, which is linked via LSOA, SOA (Northern Ireland) or datazone (Scotland), to the most recent versions of the different national Indices of Deprivation. Both the patient-level and practice-level IMD contains quintiles of deprivation (1=least deprived, 5=most deprived).

***Denmark***

In Denmark (population 5.7 million inhabitants), there is a long tradition of registering health and social data about the population in nationwide registries. For this study, we used information recorded in the Civil Registration System [4], the Danish Cancer Registry [5], the Danish Registry of Causes of Death [6], the Danish National Patient Registry [7], the Danish National Prescription Registry [8], and the Danish Education Registry [9].

The Civil Registration System [4] includes computerised data on demographics, address, vital statistics, civil status, and identifiers of close relatives (spouse, children and parents) for the entire Danish population since 1968. The Civil Registration System assigns a unique personal registration number to all Danish residents, which is used across the public sector to record information. This number facilitated linkage of all data sources used in our study. We used the Civil Registration System to identify the study population, partners, and vital status.

The Danish Cancer Registry [5] has recorded information on all incident cancers in Denmark since 1943, including information on cancer stage at time of diagnosis. During the study period, diagnoses were coded according to ICD-10 and the International Classification of Diseases for Oncology, Version 3, for topography and morphology codes. Cancer cases are reported to the Registry by general practitioners, practicing specialists, and hospital departments, including departments of pathology. Notifications are also received through other Danish databases, including the Danish National Patient Registry, the Pathology Registry, and the Registry of Causes of Death. Staging has been classified according to Summary Staging (local, regional, or distant) from 1943 through 2003, and according to the TNM classification thereafter. We used the Cancer Registry to identify all incident diagnoses of melanoma. A validation study has reported that, compared with pathology reports, the positive predictive value of melanoma diagnoses in the Registry is 97% [10] Sensitivity was estimated at 90% compared with the Pathology Registry. For stage, we used the Summary Staging classification together with the TNM, as follows: localised (TNM: T1–4, N0, M0), regional (TNM: T1–4, N1–3, M0), distant (TNM: T1–4, N1–3, M1), and unknown/missing.

The Danish Registry of Causes of Death [6] established in 1943, includes information on causes of death reported on the death certificate. We used the Danish Registry of Causes of Death to identify deaths from melanoma. The quality of cause of death reporting is unknown, because of sparse validation studies and a low autopsy rate (below 10%). A 1978 study showed that the quality was highly dependent on the cause of death, with very good correspondence for cancer deaths (positive predictive value = 97%, completeness = 92%). Misclassification is caused mainly by inaccuracies in the sequence of events leading to death. In our definition of melanoma-specific mortality, we therefore included deaths for which melanoma was coded as an underlying or a contributory cause of death.

The Danish National Patient Registry [7] is a hospital registry established in 1977. It provides complete nationwide coverage of non-psychiatric inpatient contacts since 1978 and of psychiatric inpatient stays, outpatient specialty clinic (ambulatory) visits, and emergency room contacts since 1995. Each contact is registered with information on dates of admission and discharge or start and end of outpatient follow-up, the primary diagnosis (main reason for contact), optional secondary (contributory) diagnoses, surgical procedures, and certain non-surgical treatments and examinations. Diseases are recorded at discharge, outpatient contact, or surgery by the treating physician using the ICD-8 through 1993 and the ICD-10 thereafter. We used the Danish National Patient Registry to identify comorbidities among the members of the study cohorts and deceased partners.

The Danish National Prescription Registry [8] provides information on prescription drugs dispensed at Danish pharmacies since 1995, including information on the date the prescription was dispensed and the Anatomical Therapeutic Chemical code, the number of packets/units, numerical strength, and formulation of the drug. We used the Danish National Prescription Registry to identify certain comorbidities among members of the study cohorts and deceased partners.

The Population Education Registry [9] includes information on the highest educational level attained by residents. It is based on administrative records from educational institutions and is supplemented with self-reported information for persons who completed their education before 1974 and for immigrants schooled outside Denmark. In 2007, 3% of ethnic Danes born in the 1945-1990 period had missing data. This number was higher for immigrants (up to 15%). It may be unreliable in younger people who are less likely to have attained their highest education level.

References:

1. Herrett E, Gallagher AM, Bhaskaran K, et al. Data Resource Profile: Clinical Practice Research Datalink (CPRD). Int J Epidemiol 2015;**44**(3):827-36
2. NHS Digital. Hospital Episode Statistics (HES) 2019 [Available from: [https://digital.nhs.uk/data-and-information/data-tools-and-services/data-services/hospital-episode-statistics.] Accessed 12 July 2019](https://digital.nhs.uk/data-and-information/data-tools-and-services/data-services/hospital-episode-statistics.%5d%20Accessed%2012%20July%202019).

Department for Communities and Local Government. The English Indices of Deprivation 2015 - Frequently Asked Questions (FAQs). 2015. [Available from: <http://www.communities.gov.uk/publications/corporate/statistics/indices2010technicalreport>.] Accessed 30 Aug 2019.

1. Schmidt M, Pedersen L, Sorensen HT. The Danish Civil Registration System as a tool in epidemiology. Eur J Epidemiol 2014;29(8):541-9
2. [Gjerstorff ML](https://www.ncbi.nlm.nih.gov/pubmed/?term=Gjerstorff%20ML%5BAuthor%5D&cauthor=true&cauthor_uid=21775350). The Danish Cancer Registry. [Scand J Public Health.](https://www.ncbi.nlm.nih.gov/pubmed/21775350) 2011;39(7 Suppl):42-5.
3. Helweg-Larsen K. The Danish Register of Causes of Death. Scand J Public Health. 2011;39(7 Suppl):26–9
4. Schmidt M, Schmidt SA, Sandegaard JL, et al. The Danish National Patient Registry: a review of content, data quality, and research potential. Clin Epidemiol 2015;7:449-90
5. Pottegard A, Schmidt SAJ, Wallach-Kildemoes H, et al. Data Resource Profile: The Danish National Prescription Registry. Int J Epidemiol 2017;46(3):798-98f
6. Jensen VM, Rasmussen AW. Danish Education Registers. Scand J Public Health 2011;39(7 Suppl):91-4
7. Pedersen SA, Schmidt SAJ, Klausen S, et al. Melanoma of the Skin in the Danish Cancer Registry and the Danish Melanoma Database: A Validation Study. Epidemiology 2018;29(3):442-47.

# **Supplementary material method 2. Partner algorithms.**

In the UK, we used an algorithm to identify partners based on the family number in the Clinical Practice Research Datalink (CPRD). This data item identifies people in a practice who are living in the same household or who otherwise are associated. First, we identified the entire CPRD population with an acceptable standard (i.e. a patients’ record has met certain quality standards). For each practice, we identified the up-to-standard date (i.e. date of practice meeting CPRD quality control standards). In the algorithm, the earliest of up-to-standard and study start date was defined as the study initiation date. We identified the household composition (*i.e.*, family numbers registered) and restricted the analysis to persons who were alive and registered with the practice on the study initiation date. We excluded persons whose family practice number was not linked to any other patient (assumed to be single). To avoid misclassification based on inconsistent use of the family number (*e.g.*, for persons living in the same flat block or nursing home), we did not consider cohabitees to be potential partners in cases in which the same family number was used for >10 persons. For each household, we identified couples of the opposite sex, with an age gap of ≤10 years and with no younger adult present in the household aged within 15 years of either member of the couple. We excluded couples in which both individuals were <40 years old (i.e. the minimum age of study cohort was 30 years), based on the assumption that cohabitation among friends (*e.g.*, in a flat or student dorm) and within a family is more common at younger age. Similarly, to avoid including possibly non-associated persons, we also excluded couples where both individuals were 95 years or older (*e.g.*, nursing home residents) or where one of the members of a couple had any code indicating residence in a communal establishment before the study initiation date or current practice registration date, whichever was later.

In Denmark, we identified partners using an algorithm provided by Statistics Denmark, the central agency for Danish statistics. This agency collects, processes, and publishes information on Danish society [1]. The algorithm utilises the detailed information available in the Danish Civil Registration System, including civil status, demographics, exact address, and the unique personal identifiers of a person’s spouse, parents, and children, which can be used to deduce close kinship. The algorithm classifies two persons as partners if they:

(1) are married (including same-sex marriage [previously referred to as “registered partnership”]);

(2) live at the exact same address and have at least one cohabitating child together; or

(3) live at the same address, have no cohabitating common children (except stepchildren), no other adults live at the address, and if the two persons are of opposite sex, have an age difference of less than 15 years, and are not closely related according to kinship data in the Danish Civil Registration System.

Because of the detailed data recorded in the Civil Registration System, we were not concerned about using less strict criteria (*e.g.*, a broader age gap) than in the UK. Furthermore, partner status is updated annually (on 1 January), which allowed us to identify first-time bereavement with greater certainty in Denmark.

Reference:

1. Danmarks Statistik. FAMILIE_TYPE 2019 [Available from: <https://www.dst.dk/da/Statistik/dokumentation/Times/forebyggelsesregistret/familie-type> accessed 12 July 2019.

# **Supplementary material method 3. Code lists used to define partners’ risk of death, outcomes, and other covariates in Denmark.**

**Code list of variables used to define partners’ risk of death**

| *Score 1* | ICD-8 | ICD-10^a^ | ATC |
| --- | --- | --- | --- |
| Myocardial infarction | ”410” | ”DI21” ”DI22” ”DI23” |  |
| Congestive heart failure | ”42709” ”42710” ”42711” ”42719” ”42899” ”78249” | ”DI50” ”DI110” ”DI130” ”DI132” |  |
| Peripheral vascular disease | ”440” ”441” ”442” ”443” ”444” ”445” | ”DI70” ”DI71” ”DI72” ”DI73” ”DI74” ”DI77” |  |
| Cerebrovascular disease | ”430” ”431” ”432” ”433” ”434” ”435” ”436” ”437” ”438” | ”DI60” ”DI61” ”DI62” ”DI63” ”DI64” ”DI65” ”DI66” ”DI67” ”DI68” ”DI69” ”DG45” ”DG46” |  |
| Dementia | “29009” “29010” “29011” “29018” “29019” “29309” | “DF00” “DF01” “DF02” “DF03” “DF051” “DG30” |  |
| Chronic pulmonary disease | “490” “491” “492” “493” “515” “516” “517” “518” | “DJ40” “DJ41” “DJ42” “DJ43” “DJ44” “DJ45” “DJ46” “DJ47” “DJ60” “DJ61” “DJ62” “DJ63” “DJ64” “DJ65” “DJ66” “DJ67” “DJ684” “DJ701” “DJ703” “DJ841” “DJ920” “DJ961” “DJ982” “DJ983” |  |
| Connective tissue disease | ”712” ”716” ”734” ”446” ”13599” | ”DM05” ”DM06” ”DM08” ”DM09” ”DM30” ”DM31” ”DM32” ”DM33” ”DM34” ”DM35” ”DM36” ”DD86” |  |
| Ulcer disease | “53091” “53098” “531” “532” “533” “534” | “DK221” “DK25” “DK26” “DK27” “DK28” |  |
| Mild liver disease | “571” “57301” “57304” | “DB18” “DK700” “DK701” “DK702” “DK703” “DK709” “DK71” “DK73” “DK74” “DK760” |  |
| Diabetes types 1 and 2^b^ | “249” “250” | “DE10” “DE11” “DE12” “DE13” “DE14” “DH360” “DO24” (excluding “DO244”) | “A10A” “A10B” (excluding “A10BE01”) “B04AX07” “C10AX04” |
| *Score 2* |  |  |  |
| Hemiplegia | “344” | “DG81” “DG82” |  |
| Moderate to severe renal disease | “403” “404” “580” “581” “582” “583” “584” “59009” “59319” “75310” 75311” “75319” “75320” “792” | “DI12” “DI13” “DN00” “DN01” “DN02” “DN03” “DN04” “DN05” “DN07” “DN11” “DN14” “DN17” “DN18” “DN19” “DQ61” |  |
| Diabetes with end organ-damage | “24901” “24902” “24903” “24904” “24905” “24908” “25001” “25002” “25003” “25004” “25005” “25008” | “DE102” “DE103” “DE104” “DE105” “DE106” “DE107” “DE108” “DE112” “DE113” “DE114” “DE115” “DE116” “DE117” “DE118” “DE142” “DE143” “DE144” “DE145” “DE146” “DE147” “DE148” |  |
| Any tumor | ”140” ”141” ”142” ”143” ”144” ”145” ”146” ”147” ”148” ”149” ”150” ”151” ”152” ”153” ”154” ”155” ”156” ”157” ”158” ”159” ”160” ”161” ”162” ”163” ”164” ”165” ”166” ”167” ”168” ”169” ”170” ”171” ”172” ”173” ”174” ”175” ”176” ”177” ”178” ”179” ”180” ”181” ”182” ”183” ”184” ”185” ”186” ”187” ”188” ”189” ”190” ”191” ”192” ”193” ”194” | ”DC00” ”DC01” ”DC02” ”DC03” ”DC04” ”DC05” ”DC06” ”DC07” ”DC08” ”DC09” ”DC10” ”DC11” ”DC12” ”DC13” ”DC14” ”DC15” ”DC16” ”DC17” ”DC18” ”DC19” ”DC20” ”DC21” ”DC22” ”DC23” ”DC24” ”DC25” ”DC26” ”DC27” ”DC28” ”DC29” ”DC30” ”DC31” ”DC32” ”DC33” ”DC34” ”DC35” ”DC36” ”DC37” ”DC38” ”DC39” ”DC40” ”DC41” ”DC42” ”DC43” ”DC44” ”DC45” ”DC46” ”DC47” ”DC48” ”DC49” ”DC50” ”DC51” ”DC52” ”DC53” ”DC54” ”DC55” ”DC56” ”DC57” ”DC58” ”DC59” ”DC60” ”DC61” ”DC62” ”DC63” ”DC64” ”DC65” ”DC66” ”DC67” ”DC68” ”DC69” ”DC70” ”DC71” ”DC72” ”DC73 ”DC74” ”DC75” |  |
| Leukemia | ”204” ”205” ”206” ”207” | ”DC91” ”DC92” ”DC93” ”DC94” ”DC95” |  |
| Lymphoma | ”200” ”201” ”202” ”203” ”27559” | ”DC81” ”DC82” ”DC83” ”DC84” ”DC85” ”DC88” ”DC90” ”DC96” |  |
| *Score 3* |  |  |  |
| Moderate to severe liver disease | “07000” “07002” “07004” “07006” “07008” “57300” “45600” “45601” “45609” | “DB150” “DB160” “DB162” “DB190” “DK704” “DK72” “DK766” “DI85” |  |
| *Score 6* |  |  |  |
| Metastatic solid tumor | ”195” ”196” ”197” ”198” ”199” | ”DC76” ”DC77” ”DC78” ”DC79” ”DC80” |  |
| Acquired immune deficiency syndrome | “07983” | “DB21” “DB22” “DB23” “DB24” |  |
| *Age scores*  1 point: 50-59 years  2 point: 60-69 years  3 points: 70-79 years  4 points: 80-89 years  5 points: 90-99 years |  |  |  |
| Terminal disease (recorded for partners before death) |  | “DZ515” “DZ756”  From Danish National Patient Registry we also included supplementary codes “ZNAC14” “ZPZA05. | Prescription record for a terminal patient (that is, variable PATT with value “99”) |

Note: All subcodes were included unless otherwise stated; all types of contacts (inpatient, outpatient and emergency) were considered. We used the codes for all variables that were assigned on the hospital discharge date, except for terminal disease, as this is coded shortly before death.

^a^All ICD-10 codes coded in the National Patient Registry begins with “D”. For example, DI21 represents I21.

^b^Diabetes is identified as any previous diabetes diagnosis or two or more antidiabetic prescriptions. Women identified solely by monotherapy with metformin (A10BA02) at ages 20–39 years are not considered, because they may have had polycystic ovarian syndrome as the indication for their prescription.

**Outcome definitions**

| **Outcome variable** | **ICD-10** | **Notes** |
| --- | --- | --- |
| Melanoma (Danish Cancer Registry) | “C43” | First-time diagnosis in the Danish Patient Registry. |
| Melanoma mortality (Cause of Death Registry) |  | Cause-specific death, with melanoma registered either as the underlying or immediate cause of death. |

Note: All subcodes were included unless otherwise stated; all types of contacts (inpatient, outpatient and emergency) were considered. The codes for all variables were assigned on the admission date.

**Covariate definitions (measured prior to index date)**

| **Variable** | **Notes** |
| --- | --- |
| Highest attained education | Defined based on variable hfaudd as short (hfaudd=10), medium (hfaudd=20–39), or long (hfaudd=40–89). See Method S2 for more detail. |
| Study participant’s (conventional/non-age adjusted) Charlson Comorbidity Index score (grouped as low=0, intermediate=1–2, and high≥3). | Same coding as for risk of partners’ deaths described above, but without age points and considering all records 5 years prior to index date (*e.g*., including the month prior to the index date). |

Note: All subcodes were included unless otherwise stated; all types of contacts (inpatient, outpatient and emergency) were considered. The codes for all variables were assigned on the admission date.

# **Supplementary material method 4. Identification of melanoma mortality.**

In the UK, we identified melanoma mortality using ICD-9 (172) and ICD-10 (C43) codes recorded in ONS death data. We required that individuals had linked ONS data available and their melanoma diagnosis had to be after 2 January 1998 (when ONS data became available). In Denmark, we identified deaths recorded with ICD-8 or ICD-10 codes for melanoma in the Registry of Causes of Death. In both countries, we considered all deaths to be melanoma-specific if they were registered either as the underlying, or a contributory, cause of death.

# **Supplementary material method 5. Details on covariates.**

We used the original Charlson Comorbidity Index (CCI) score [1], categorised as: low (0 point), intermediate (1–2 points), or high (≥3 points) to classify comorbidity burden on the follow-up start date (*i.e.*, matching date in the Study 1 *incidence analysis* and melanoma diagnosis date in the Study 2 *mortality analysis*). In calculating CCI scores, we excluded all skin cancer diagnoses when calculating CCI scores to minimise the risk of including outcome (i.e. melanoma diagnosis) as a covariate due to potential misclassification of melanoma and other skin cancers. In the UK, we identified BMI (categories: <18.5, 18.5–24.9, 25–29.9, and ≥30 kg/m^2^), alcohol consumption (categories: current drinker, ex-drinker, non-drinker) and smoking status (categories: current smoker, ex-smoker, non-smoker). We used IMD score, stratified by quintiles, as a measure of socioeconomic deprivation in the UK. When individual-level IMD status was missing (40.4%), we used the practice-level IMD. In Denmark, we used education duration as a proxy for socioeconomic status, categorised as short (7–10 years), medium (11–12 years), or long (≥13 years).

We hypothesised that the level of stress associated with bereavement may depend on whether a partner’s death was unexpected. Therefore, we stratified the estimates by the degree to which the partner’s death might be considered unexpected based on level of comorbidity. We estimated the age-adjusted CCI score for the deceased partner based on comorbidities recorded up to a month before their death. The CCI assigns 0 to 6 points to a range of chronic diseases according to the ability of each to predict death, with additional points applied according to age. We classified the risk of partner death as low (0­–3 points), intermediate (4–6 points) or high (≥7 points). As an alternative measure, we identified presence of terminal disease among partners recorded before the date of death.

Reference:

1. Charlson M, Szatrowski TP, Peterson J, Gold J. Validation of a combined comorbidity index. J Clin Epidemiol 1994;47(11):1245-51.

# **Supplementary material figure 1. Illustration of follow-up in the melanoma incidence analysis.**

**
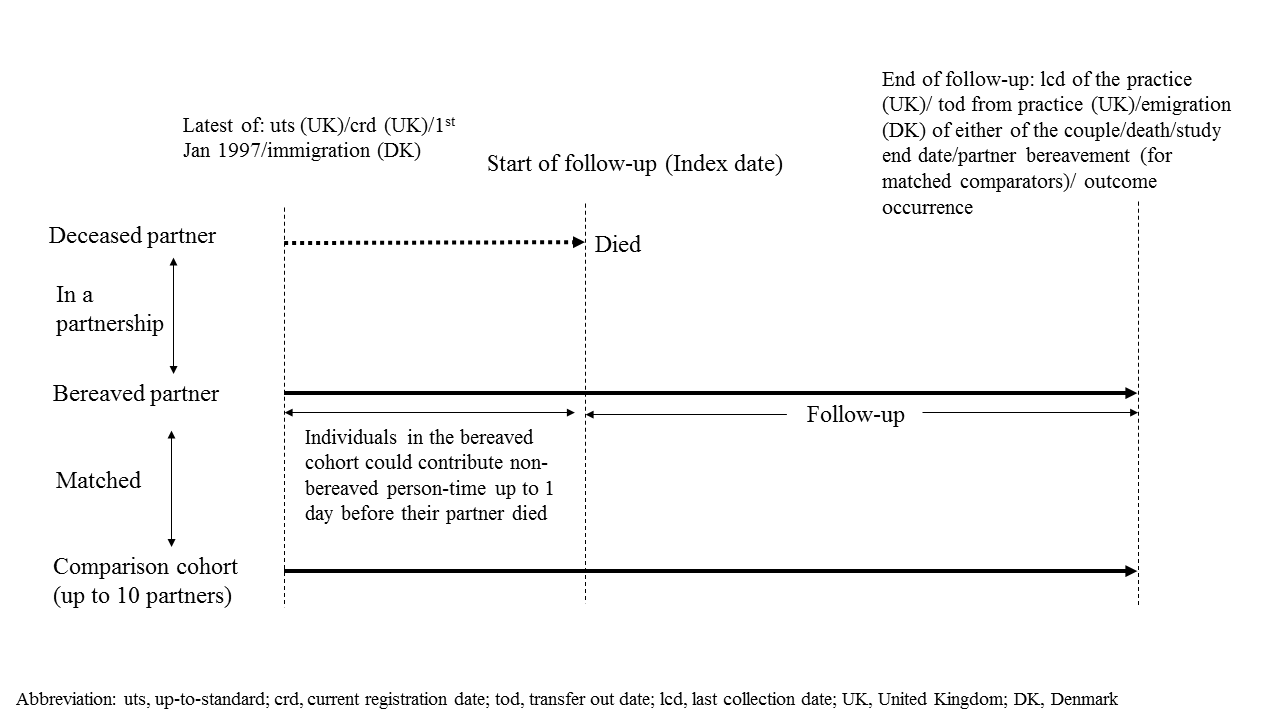
**

# **Supplementary material figure 2. Illustration of follow-up in the melanoma mortality analysis.**

Start of study

End of study

**Key:**

Partner bereavement

Date of melanoma diagnosis (start of follow-up)

Censored*

Death from melanoma

Bereaved

Bereaved

Bereaved

Non-bereaved

Non-bereaved

*We censored follow-up on the last date of data collection from the practice (UK), transfer out of practice of either member of the couple (UK), emigration of either member of the couple (Denmark), death, or study end date.

# **Supplementary material figure 3. Assessment of the assumption of proportional hazards.**


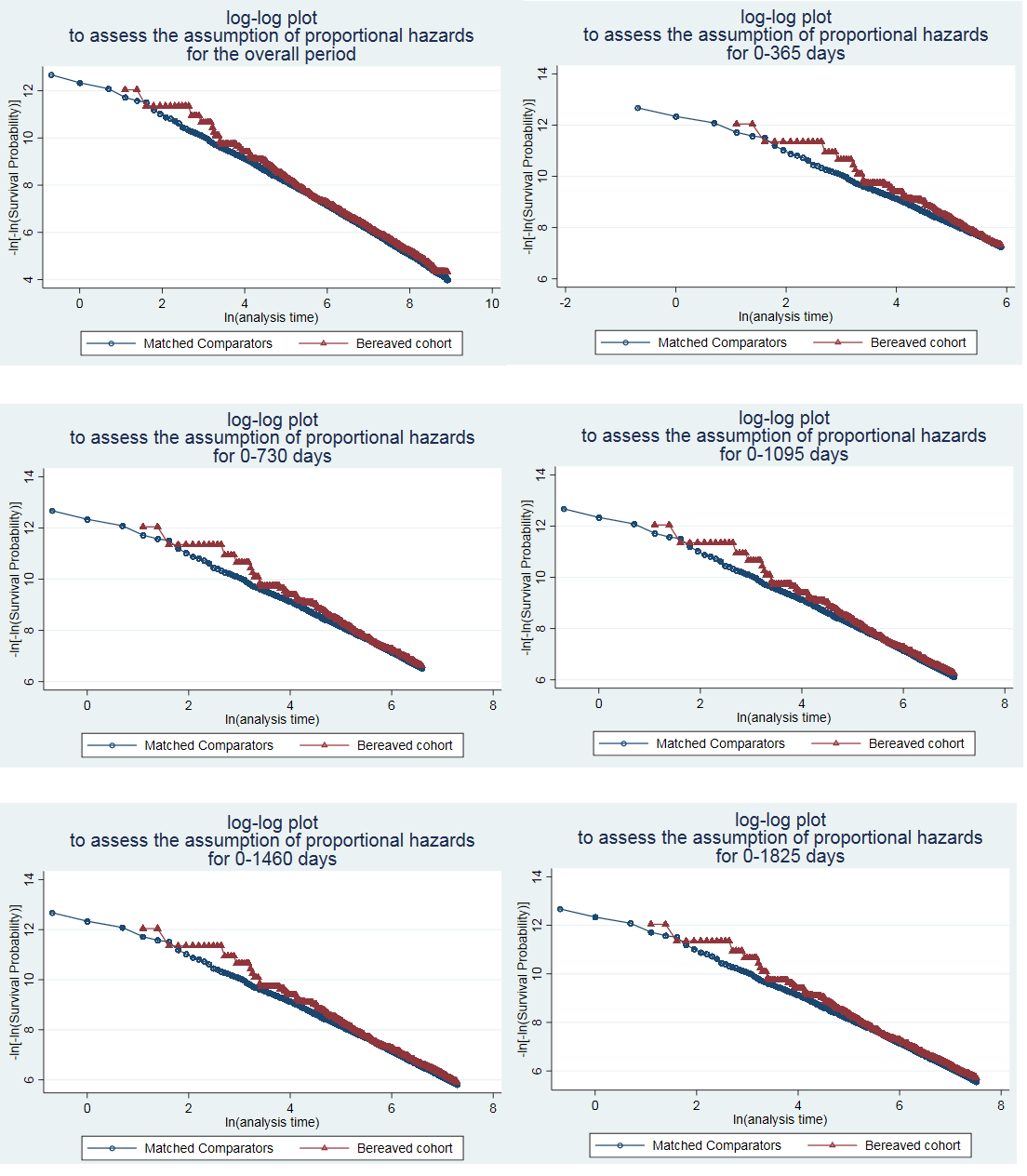


Supplementary Figure 3.1 Log-log plots assessing the assumption of proportional hazards in the analysis of the association between partner bereavement and melanoma in the UK.


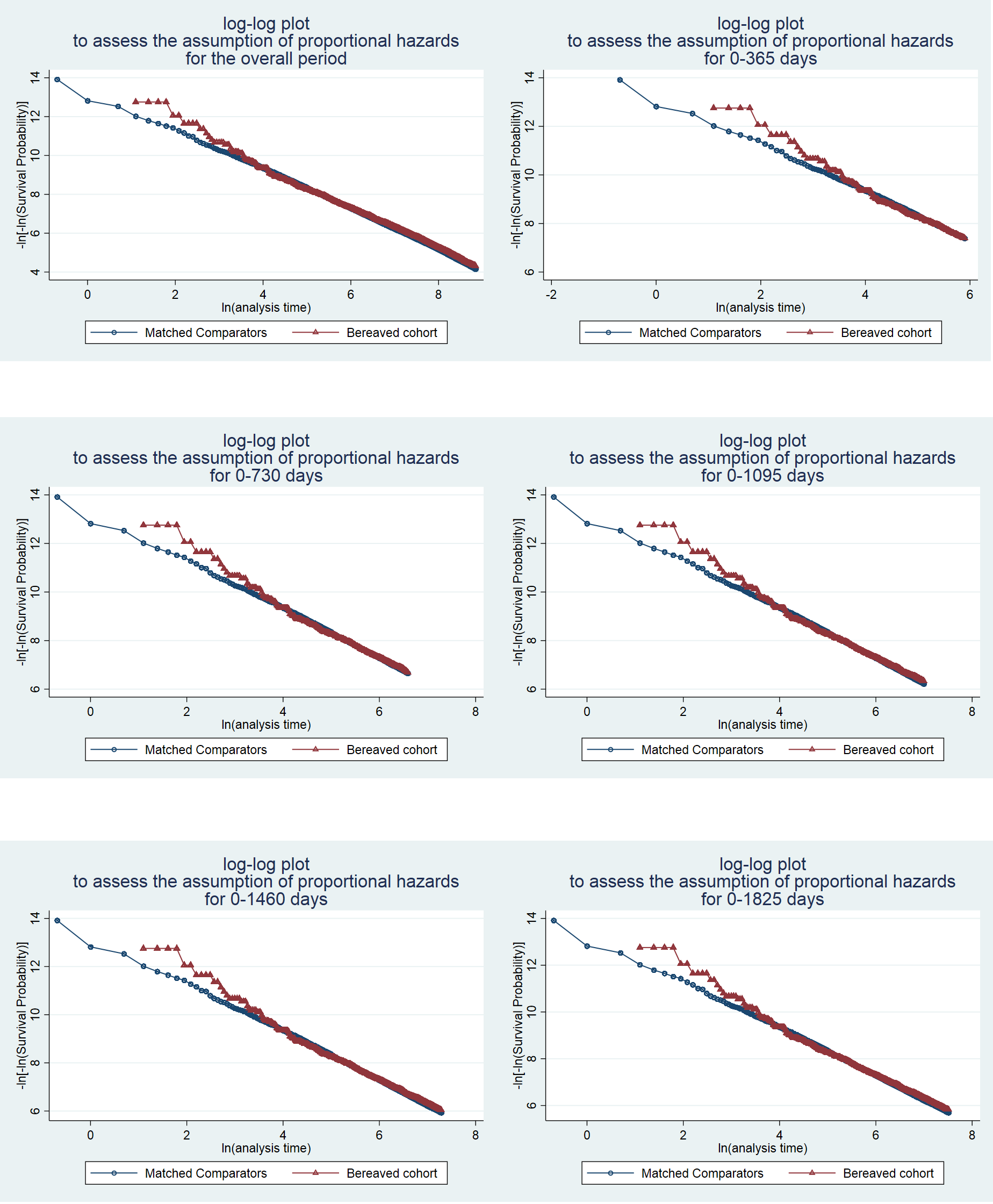


Supplementary Figure 3.2. Log-log plots assessing the assumption of proportional hazards in the analysis of the association between partner bereavement and melanoma in Denmark.

# **Supplementary material table 1. Results of stratifying follow-up time since partner bereavement in the melanoma incidence analysis.**

|  | **UK study** |  | **Danish study** |  |
| --- | --- | --- | --- | --- |
|  | **Unadjusted hazard ratio**  **(95% CI)** |  | **Unadjusted hazard ratio**  **(95% CI)** |  |
| 0-1 year | 0.93 (0.76-1.14) |  | 0.99 (0.86-1.15) |  |
| 1-2 years | 0.82 (0.65-1.02) |  | 0.96 (0.83-1.12) |  |
| 2-3 years | 0.83 (0.65-1.07) | p=0.30 | 0.76 (0.64-0.91) | p=0.15 |
| 3-4 years | 0.97 (0.76-1.25) |  | 0.90 (0.76-1.07) |  |
| 4-5 years | 0.63 (0.46-0.85) |  | 0.78 (0.65-0.94) |  |
| 5+ years | 0.86 (0.74-0.99) |  | 0.88 (0.82-0.95) |  |

Abbreviation: CI, confidence interval

# **Supplementary material table 2. List of sensitivity analyses.**

| **Sensitivity analysis** | **Justification** | **UK** | **Denmark** |
| --- | --- | --- | --- |
| **Melanoma incidence analysis** | | | |
| 1. Repeated main analysis restricted to individuals with at least 5 years of registration history in data sources prior to the index date. (See Table S7) | General practitioners tend to record prevalent conditions when new patients register.^1^ By including individuals who recently registered with their general practitioner, we risk misclassifying prevalent melanoma as incident diagnoses (*i.e.*, outcomes) in the study. This sensitivity analysis aimed to limit such misclassification. | √ | √ |
| 1. Repeated main analysis restricted to individuals eligible for linkage to Hospital Episode Statistics/Office for National Statistics death registration data and those who had follow-up start date after Office for National Statistics linkage coverage start date (i.e. 2 January 1998). (See Table S8) | Hospital admission records and more accurate death dates are available in linked HES and ONS data, providing more complete and accurate exposure and outcomes data for this subset of patients. | √ |  |
| 1. Repeated main analysis without censoring persons from the comparison cohort at partner bereavement, at transfer of their partner out of the medical practice (UK), or at emigration of partner (Denmark) (*post-hoc*). (See Table S9) | To examine data as an intention-to-treat analysis, in order to limit potential bias due to informative censoring by death or loss to follow-up of partners. | √ | √ |
| 1. Redefined a cohort using the same matching criteria without replacement with a matching ratio of 1:1 (*post-hoc*). (See Table S10) | To explore whether the clusters of subjects affect the standard errors of the main analysis, as a person in the comparison cohort could be matched to more than one bereaved individual across the cohort in the main analysis. | √ |  |
| 1. Repeated main analysis after adding the end of partnership (separation/divorce) as a censoring criterion. (See Table S11) | To examine the impact of a change in partnership status that removed the risk of exposure. |  | √ |
| 1. Repeated main analysis after including only histologically verified diagnoses in the outcome definition. (See Table S12) | To increase the validity of the outcome. |  | √ |
| **Melanoma mortality analysis** | | | |
| 1. Investigated the risk of all-cause mortality as an additional outcome (*post-hoc*). (See Table S15) | All-cause mortality is an important outcome for understanding the prognosis of patients with melanoma. All-cause mortality also served as a secondary outcome to compare findings for melanoma mortality among those who could be linked to Office for National Statistics death registration data. | √ | √ |
| 1. Repeated the analysis for all-cause mortality, not limited to subjects with linkage to Office for National Statistics death registration data (*post-hoc*). (See Tables S18-S19) | As death data are also available in the CPRD, we did not restrict the cohort to those with linkage to Office for National Statistics death registration data. This was an additional analysis to assess the sensitivity of the results for all-cause mortality. | √ |  |
| 1. Repeated main analysis excluding those who experienced bereavement prior to/on the date of melanoma diagnosis. (See Table S20) | The effects of bereavement are unlikely to remain unchanged over time. Including persons who were bereaved prior to melanoma diagnosis without considering the duration of bereavement might introduce heterogeneity of exposure effects. This sensitivity analysis aimed to retain only those who experienced bereavement during follow-up to minimise heterogeneity. | √ | √ |
| 1. Repeated main analysis excluding patients who lost their partner or were no longer together with their partner 3 years prior to melanoma diagnosis (*post-hoc*). (See Table S21) | To examine the impact of the change in partnership status resulting in persons not being at risk of exposure anymore. |  | √ |
| 1. Repeated main analysis adding the end of partnership (separation/divorce) as a censoring criterion. (See Table S22) | To examine any impact of a change in partnership status that removed the risk of exposure. |  | √ |
| 1. Repeated main analysis censoring the follow-up at emigration or end of partnership, and excluding patients who experienced these prior to melanoma diagnosis (*post-hoc*). (See Table S23) | To examine any impact of a change in partnership status that removed the risk of exposure and to examine the impact of loss of follow-up due to emigration. |  | √ |
| 1. Repeated main analysis after excluding patients with a melanoma diagnosis, that was not histologically verified. (See Table S24) | To increase the validity of the outcome. |  | √ |

Reference

1. Lewis JD, Bilker WB, Weinstein RB, Strom BL. The relationship between time since registration and measured incidence rates in the General Practice Research Database. Pharmacoepidemiol Drug Saf 2005;14(7):443-51.

# **Supplementary material table 3. Association between partner bereavement and diagnosis of incident melanoma, overall & by time since the follow-up start date.**

| **Time since follow-up start date** | **Bereaved cohort** | | | **Matched comparators** | | | **Unadjusted HR (95% CI)^a^** | **Adjusted HR (95% CI)^b^** | **Fully adjusted HR (95% CI)^c^** |
| --- | --- | --- | --- | --- | --- | --- | --- | --- | --- |
|  | **Number of events** | **Person-years at risk** | **Rate per 1,000** | **Number of events** | **Person-years at risk** | **Rate per 1,000** |  |  |  |
| **UK** | | | | | | | | | |
| Entire follow-up | 620 | 905,281 | 0.68 | 6430 | 8,137,953 | 0.79 | 0.85 (0.78-0.93) | 0.85 (0.78-0.93) | 0.86 (0.79-0.94) |
| 0–1 year | 104 | 155,818 | 0.67 | 1052 | 1,479,290 | 0.71 | 0.93 (0.76-1.14) | 0.92 (0.75-1.13) | 0.93 (0.75-1.15) |
| 0–2 year | 191 | 288,900 | 0.66 | 2002 | 2,737,961 | 0.73 | 0.87 (0.75-1.01) | 0.87 (0.75-1.01) | 0.88 (0.76-1.04) |
| 0-3 year | 260 | 402,950 | 0.65 | 2781 | 3,801,859 | 0.73 | 0.86 (0.76-0.98) | 0.86 (0.76-0.98) | 0.88 (0.77-1.01) |
| 0-4 year | 335 | 500,318 | 0.67 | 3487 | 4,695,873 | 0.74 | 0.88 (0.79-0.99) | 0.89 (0.79-0.99) | 0.91 (0.81-1.03) |
| 0-5 year | 382 | 582,924 | 0.66 | 4140 | 5,440,266 | 0.76 | 0.84 (0.76-0.94) | 0.85 (0.76-0.94) | 0.87 (0.78-0.97) |
| **Denmark** | | | | | | | | | |
| Entire follow-up | 1,667 | 2,552,711 | 0.65 | 16,166 | 22,027,622 | 0.73 | 0.89 (0.84-0.93) | 0.89 (0.84-0.93) | 0.89 (0.84-0.94) |
| 0–1 year | 203 | 329,407 | 0.62 | 1,944 | 3,137,524 | 0.62 | 0.99 (0.86-1.15) | 0.99 (0.86-1.15) | 0.97 (0.83-1.13) |
| 0–2 year | 391 | 629,025 | 0.62 | 3,826 | 5,931,262 | 0.65 | 0.98 (0.88-1.08) | 0.98 (0.88-1.08) | 0.96 (0.86-1.08) |
| 0-3 year | 537 | 901,143 | 0.60 | 5,566 | 8,411,041 | 0.66 | 0.91 (0.83-0.99) | 0.91 (0.83-0.99) | 0.90 (0.82-0.99) |
| 0-4 year | 686 | 1,146,833 | 0.60 | 7,021 | 10,598,701 | 0.66 | 0.91 (0.84-0.98) | 0.91 (0.84-0.98) | 0.90 (0.83-0.98) |
| 0-5 year | 811 | 1,367,420 | 0.59 | 8,395 | 12,521,393 | 0.67 | 0.89 (0.82-0.95) | 0.89 (0.82-0.95) | 0.88 (0.81-0.95) |

Abbreviations: HR, hazard ratio; CI, confidence interval

^a^Computed using Cox regression stratified by matched set to account for the matching variables of age, sex, county of residence (in Denmark) and general practice (in the UK).

^b^Adjusted for Charlson Comorbidity Index score.

^c^Complete-case analysis was used to handle missing data in the fully adjusted model. Notably, the number of events, person-years at risk and rate per 1000 person-years in the bereaved and matched comparator cohorts presented in this table were calculated for the full cohort in the unadjusted and adjusted models only. Adjusted additionally for smoking status, body mass index, alcohol consumption and deprivation status in the UK. The total number of bereaved and comparison persons was 150,547 and 1,306,385, respectively, after excluding those with missing values for body mass index, alcohol consumption and smoking status. Adjusted additionally for education duration in Denmark. The total number of bereaved and comparison persons was 300,686 and 2,857,617, respectively, after excluding those with missing values for education duration.

# **Supplementary material table 4. Association between partner bereavement and diagnosis of incident melanoma, subgroup analysis.**

|  | **Bereaved cohort** | | | | **Matched comparators** | | | | **Unadjusted HR (95% CI)^a^** | **Adjusted HR**  **(95% CI)^b^** | | **Fully adjusted HR (95% CI)^c^** |
| --- | --- | --- | --- | --- | --- | --- | --- | --- | --- | --- | --- | --- |
|  | **Number of persons** | **Number of events** | **Person-years at risk** | **Rate per 1,000** | **Number of persons** | **Number of events** | **Person-years at risk** | **Rate per 1,000** |  |  |  |  |
| **UK** | | | | | | | | | | | | |
| *Age* | | | | | | | | | | | | |
| <50 | 3,081 | 13 | 25,743 | 0.50 | 30,096 | 106 | 268,689 | 0.39 | 1.40 (0.77-2.52) | 1.39 (0.77-2.52) | p=0.0043 | 1.37 (0.74-2.55) |
| 50–59 | 15,843 | 33 | 111,523 | 0.30 | 158,537 | 670 | 1,147,097 | 0.58 | 0.51 (0.36-0.73) | 0.51 (0.36-0.73) |  | 0.52 (0.36-0.75) |
| 60–69 | 39,239 | 142 | 249,220 | 0.57 | 391,003 | 1734 | 2,416,415 | 0.72 | 0.77 (0.65-0.92) | 0.77 (0.65-0.92) |  | 0.80 (0.67-0.96) |
| 70–79 | 64,000 | 268 | 346,789 | 0.77 | 630,668 | 2672 | 3,084,959 | 0.87 | 0.92 (0.81-1.05) | 0.92 (0.81-1.05) |  | 0.92 (0.80-1.06) |
| ≥80 | 47,839 | 164 | 172,005 | 0.95 | 388,956 | 1248 | 1,220,792 | 1.02 | 0.92 (0.77-1.10) | 0.93 (0.77-1.11) |  | 0.95 (0.78-1.14) |
| *Sex* | | | | | | | | | | | | |
| Female | 111,427 | 373 | 619,656 | 0.60 | 1,048,995 | 3666 | 5,353,281 | 0.68 | 0.86 (0.77-0.96) | 0.86 (0.77-0.96) | p=0.85 | 0.85 (0.76-0.96) |
| Male | 58,575 | 247 | 285,625 | 0.86 | 550,265 | 2764 | 2,784,672 | 0.99 | 0.84 (0.73-0.96) | 0.84 (0.73-0.97) |  | 0.88 (0.77-1.02) |
| *Risk of partner death by ACCI^d^* | | | | | | | | | | | | |
| Low | 26,131 | 96 | 179,554 | 0.53 | 258,124 | 1213 | 1,774,496 | 0.68 | 0.76 (0.61-0.94) | 0.76 (0.61-0.94) | p=0.18 | 0.81 (0.65-1.01) |
| Intermediate | 66,309 | 237 | 364,871 | 0.65 | 625,184 | 2614 | 3,239,317 | 0.81 | 0.81 (0.70-0.93) | 0.81 (0.70-0.93) |  | 0.82 (0.71-0.95) |
| High | 77,562 | 287 | 360,855 | 0.80 | 715,952 | 2603 | 3,124,139 | 0.83 | 0.93 (0.82-1.05) | 0.93 (0.82-1.06) |  | 0.93 (0.81-1.06) |
| *Risk of partner death by terminal disease^e^* | | | | | | | | | | | | |
| Yes | 43,743 | 148 | 197,033 | 0.75 | 418,565 | 1453 | 1,822,786 | 0.80 | 0.91 (0.76-1.08) | 0.91 (0.77-1.08) | p=0.38 | 0.91 (0.75-1.09) |
| No | 126,259 | 472 | 708,248 | 0.67 | 1,180,695 | 4977 | 6,315,166 | 0.79 | 0.83 (0.75-0.92) | 0.83 (0.75-0.92) |  | 0.85 (0.77-0.94) |
| **Denmark** | | | | | | | | | | | | |
| *Age* | | | | | | | | | | | | |
| <50 | 23,956 | 75 | 243,495 | 0.31 | 238,640 | 1,020 | 2,424,755 | 0.42 | 0.74 (0.59-0.94) | 0.74 (0.58-0.93) |  | 0.77 (0.60-0.97) |
| 50–59 | 45,143 | 228 | 438,375 | 0.52 | 449,727 | 2,519 | 4,247,876 | 0.59 | 0.87 (0.76-1.00) | 0.87 (0.76-1.00) |  | 0.90 (0.78-1.03) |
| 60–69 | 89,214 | 496 | 744,325 | 0.67 | 887,777 | 4,942 | 6,756,809 | 0.73 | 0.91 (0.83-1.00) | 0.91 (0.83-1.00) | p=0.33 | 0.92 (0.84-1.01) |
| 70–79 | 114,708 | 581 | 794,205 | 0.73 | 1,123,948 | 5,484 | 6,358,183 | 0.86 | 0.86 (0.79-0.94) | 0.86 (0.78-0.94) |  | 0.85 (0.77-0.93) |
| ≥80 | 72,894 | 287 | 332,310 | 0.86 | 619,696 | 2,201 | 2,239,999 | 0.98 | 0.97 (0.85-1.11) | 0.97 (0.85-1.11) |  | 0.95 (0.80-1.12) |
| *Sex* | | | | | | | | | | | | |
| Female | 231,022 | 1,061 | 1,797,216 | 0.59 | 2,214,531 | 9,429 | 14,683,758 | 0.64 | 0.89 (0.83-0.95) | 0.89 (0.83-0.95) |  | 0.89 (0.83-0.95) |
| Male | 114,893 | 606 | 755,494 | 0.80 | 1,105,257 | 6,737 | 7,343,864 | 0.92 | 0.88 (0.81-0.96) | 0.88 (0.81-0.96) | p=0.91 | 0.88 (0.80-0.97) |
| *Risk of partner death by ACCI^d^* | | | | | | | | | | | | |
| Low | 92,187 | 462 | 833,753 | 0.55 | 912,590 | 5,053 | 7,805,008 | 0.65 | 0.86 (0.78-0.94) | 0.86 (0.78-0.94) |  | 0.86 (0.78-0.95) |
| Intermediate | 165,168 | 789 | 1,144,423 | 0.69 | 1,558,810 | 7,243 | 9,377,334 | 0.77 | 0.90 (0.83-0.97) | 0.90 (0.83-0.97) | p=0.71 | 0.89 (0.82-0.97) |
| High | 88,560 | 416 | 574,535 | 0.72 | 848,388 | 3,870 | 4,845,280 | 0.80 | 0.90 (0.81-1.00) | 0.90 (0.81-1.00) |  | 0.91 (0.82-1.02) |
| *Risk of partner death by terminal disease^e^* | | | | | | | | | | | | |
| Yes | 76,293 | 336 | 467,838 | 0.72 | 748,688 | 3,217 | 4,241,300 | 0.76 | 0.92 (0.82-1.03) | 0.92 (0.82-1.03) |  | 0.92 (0.82-1.04) |
| No | 269,622 | 1,331 | 2,084,873 | 0.64 | 2,571,100 | 12,949 | 17,786,322 | 0.73 | 0.88 (0.83-0.93) | 0.88 (0.83-0.93) | p=0.46 | 0.88 (0.82-0.93) |

Abbreviations: ACCI, age-adjusted Charlson Comorbidity Index; HR, hazard ratio; CI, confidence interval

^a^Computed using Cox regression stratified by matched set to account for the matching variables of age, sex, county of residence (in Denmark) and general practice (in the UK).

^b^Adjusted for Charlson Comorbidity Index score.

^c^Complete-case analysis was used to handle missing data in the fully adjusted model. Notably, the number of events, person-years at risk and rate per 1,000 person-years in the bereaved and matched comparators cohorts presented in this table were calculated for the full cohort in the unadjusted and adjusted models only. Adjusted additionally for smoking status, body mass index, alcohol consumption and deprivation status in the UK. The total number of bereaved and comparison persons was 150,547 and 1,306,385 respectively after excluding persons with missing values for body mass index, alcohol consumption and smoking status. Adjusted additionally for education duration in Denmark. The total number of bereaved and comparison persons was 300,686 and 2,857,617 respectively after excluding persons with missing values for education duration.

^d^Age-adjusted Charlson Comorbidity Index scores were computed based on comorbidity recorded up to one month before the death of deceased partners. This index assigns 0 to 6 points to a range of chronic diseases according to their ability to predict death, with additional points given according to age.

^e^Alternative measure for predicting partners’ deaths; records for terminal disease were identified for partners at time of death.

# **Supplementary material table 5. Patterns of missingness of smoking status, body mass index, and alcohol consumption data in the melanoma incidence analysis.**

Table 5.1. Missing data on smoking status

| **N (%) with missing data** | 24,064 (1.36) |
| --- | --- |
| **Covariates** | **Odds ratio (95% CI)** |
| Outcome | 0.76 (0.26-2.22) |
| Exposure – partner bereavement | 0.91 (0.73-1.15) |
| Body Mass Index |  |
| Underweight | 1 |
| Normal weight | 0.56 (0.35-0.92) |
| Overweight | 0.70 (0.43-1.13) |
| Obese | 0.68 (0.41-1.11) |
| Deprivation status |  |
| 1 (least deprived) | 1 |
| 2 | 0.93 (0.64-1.33) |
| 3 | 0.62 (0.41-0.93) |
| 4 | 0.57 (0.37-0.87) |
| 5 (most deprived) | 0.51 (0.28-0.94) |
| Alcohol Status |  |
| Non-drinker | 1 |
| Current drinker | 1.03 (0.80-1.32) |
| Ex-drinker | 0.54 (0.39-0.75) |
| Charlson Comorbidity Index |  |
| Low (score 0) | 1 |
| Intermediate (score 1–2) | 0.72 (0.61-0.85) |
| High (score ≥3) | 0.50 (0.40-0.64) |

Abbreviations: CI, confidence interval

Conditional logistic regression was used with covariates of the binary outcome variable (incident melanoma diagnosis), the binary exposure variable (partner bereavement), body mass index, deprivation status, alcohol consumption, and the Charlson Comorbidity Index score to predict the missingness of smoking status (with observed smoking status as the reference).

Table 5.2. Missing data on Body Mass Index

| **N (%) with missing data** | 115,654 (6.54) |
| --- | --- |
| **Covariates** | **Odds ratio (95% CI)** |
| Outcome | 0.61 (0.51-0.74) |
| Exposure – partner bereavement | 1.20 (1.16-1.23) |
| Smoking status |  |
| Non-smoker | 1 |
| Current smoker | 1.08 (1.05-1.11) |
| Ex-smoker | 0.61 (0.59-0.62) |
| Deprivation status |  |
| 1 (least deprived) | 1 |
| 2 | 0.93 (0.90-0.97) |
| 3 | 0.95 (0.91-0.99) |
| 4 | 1.01 (0.96-1.06) |
| 5 (most deprived) | 1.00 (0.94-1.06) |
| Alcohol Status |  |
| Non-drinker | 1 |
| Current drinker | 0.56 (0.55-0.58) |
| Ex-drinker | 0.34 (0.32-0.35) |
| Charlson Comorbidity Index |  |
| Low (score 0) | 1 |
| Intermediate (score 1–2) | 0.67 (0.65-0.68) |
| High (score ≥3) | 0.55 (0.53-0.57) |

Abbreviations: CI, confidence interval

Conditional logistic regression was used with covariates of the binary outcome variable (incident melanoma diagnosis), the binary exposure variable (partner bereavement), smoking status, deprivation status, alcohol consumption, and the Charlson Comorbidity Index score to predict the missingness of body mass index (with observed body mass index as the reference).

Table 5.3. Missing data on alcohol consumption

| **N (%) with missing data** | 121,934 (6.89) |
| --- | --- |
| **Covariates** | **Odds ratio (95% CI)** |
| Outcome | 0.86 (0.73-1.01) |
| Exposure – partner bereavement | 1.09 (1.06-1.12) |
| Smoking status |  |
| Non-smoker | 1 |
| Current smoker | 0.91 (0.89-0.94) |
| Ex-smoker | 0.70 (0.68-0.71) |
| Deprivation status |  |
| 1 (least deprived) | 1 |
| 2 | 0.97 (0.94-1.01) |
| 3 | 1.09 (1.04-1.13) |
| 4 | 1.08 (1.04-1.13) |
| 5 (most deprived) | 1.00 (0.94-1.06) |
| Body Mass Index |  |
| Underweight | 1 |
| Normal weight | 0.58 (0.55-0.61) |
| Overweight | 0.51 (0.48-0.54) |
| Obese | 0.54 (0.51-0.57) |
| Charlson Comorbidity Index |  |
| Low (score 0) | 1 |
| Intermediate (score 1–2) | 0.85 (0.83-0.86) |
| High (score ≥3) | 0.77 (0.75-0.79) |

Abbreviations: CI, confidence interval

Conditional logistic regression was used with covariates of the binary outcome variable (incident melanoma diagnosis), the binary exposure variable (partner bereavement), smoking status, body mass index, deprivation status, and the Charlson Comorbidity Index score to predict the missingness of alcohol consumption (with observed alcohol consumption as the reference).

# **Supplementary material table 6. Association between partner bereavement and diagnosis of incident melanoma. Unadjusted and adjusted hazard ratios for the full cohort and the complete case cohort.**

|  | **Full cohort** | | **Complete case cohort** | | **Full cohort** | | **Complete case cohort** | |
| --- | --- | --- | --- | --- | --- | --- | --- | --- |
| **UK** | | | | | | | | |
|  | Bereaved | Matched comparators | Bereaved | Matched comparators | Bereaved | Matched comparators | Bereaved | Matched comparators |
| N | 170,002 | 1,599,260 | 150,547 | 1,306,385 | 170,002 | 1,599,260 | 150,547 | 1,306,385 |
| Time since start of follow-up | **Unadjusted HR (95% CI)^a^** | | | | **Adjusted HR (95% CI)^b^** | | | |
| Entire follow-up period | 0.85 (0.78-0.93) | | 0.84 (0.77-0.92) | | 0.85 (0.78-0.93) | | 0.84 (0.77-0.92) | |
| 0–1 year | 0.93 (0.76-1.14) | | 0.91 (0.74-1.13) | | 0.92 (0.75-1.13) | | 0.91 (0.74-1.13) | |
| 0–2 years | 0.87 (0.75-1.01) | | 0.87 (0.74-1.02) | | 0.87 (0.75-1.01) | | 0.87 (0.74-1.02) | |
| 0–3 years | 0.86 (0.76-0.98) | | 0.86 (0.75-0.99) | | 0.86 (0.76-0.98) | | 0.86 (0.75-0.99) | |
| 0–4 years | 0.88 (0.79-0.99) | | 0.89 (0.79-1.00) | | 0.89 (0.79-0.99) | | 0.89 (0.79-1.01) | |
| 0–5 years | 0.84 (0.76-0.94) | | 0.85 (0.76-0.95) | | 0.85 (0.76-0.94) | | 0.85 (0.76-0.95) | |
| **Denmark** |  | |  | |  | |  | |
|  | Bereaved | Matched comparators | Bereaved | Matched comparators | Bereaved | Matched comparators | Bereaved | Matched comparators |
| N | 345,915 | 3,319,788 | 300,686 | 2,857,617 | 345,915 | 3,319,788 | 300,686 | 2,857,617 |
| Time since start of follow-up | **Unadjusted HR (95% CI)^a^** | | | | **Adjusted HR (95% CI)^b^** | | | |
| Entire follow-up period | 0.89 (0.84-0.93) | | 0.87 (0.83-0.92) | | 0.89 (0.84-0.93) | | 0.87 (0.83-0.92) | |
| 0–1 year | 0.99 (0.86-1.15) | | 0.96 (0.82-1.12) | | 0.99 (0.86-1.15) | | 0.96 (0.82-1.12) | |
| 0–2 years | 0.98 (0.88-1.08) | | 0.95 (0.85-1.06) | | 0.98 (0.88-1.08) | | 0.95 (0.85-1.06) | |
| 0–3 years | 0.91 (0.83-0.99) | | 0.89 (0.81-0.98) | | 0.91 (0.83-0.99) | | 0.89 (0.81-0.98) | |
| 0–4 years | 0.91 (0.84-0.98) | | 0.89 (0.81-0.96) | | 0.91 (0.84-0.98) | | 0.89 (0.82-0.97) | |
| 0–5 years | 0.89 (0.82-0.95) | | 0.87 (0.80-0.94) | | 0.89 (0.82-0.95) | | 0.87 (0.80-0.94) | |

Abbreviations: HR, hazard ratio; CI, confidence interval

^a^Computed using Cox regression stratified by matched set to account for the matching variables of age, sex, county of residence (in Denmark) and general practice (in the UK).

^b^Adjusted for the Charlson Comorbidity Index score.

# **Supplementary material table 7. Association between partner bereavement and incident melanoma, sensitivity analysis restricted to patients with more than 5 years of registration history prior to the index date.**

| **UK** | | | | | | | | | |
| --- | --- | --- | --- | --- | --- | --- | --- | --- | --- |
| **Time since start of follow-up** | **Bereaved cohort (N=128,104)** | | | **Matched comparators (N=1,121,797)** | | | **Unadjusted HR (95% CI)^a^** | **Adjusted HR (95% CI)^b^** | **Fully adjusted HR (95% CI)^c^** |
|  | **Number of events** | **Person-years at risk** | **Rate per 1,000** | **Number of events** | **Person-years at risk** | **Rate per 1,000** |  |  |  |
| Entire follow-up period | 426 | 623,129 | 0.68 | 4180 | 5,195,395 | 0.80 | 0.85 (0.77-0.94) | 0.85 (0.77-0.94) | 0.86 (0.77-0.95) |
| 0–1 year | 77 | 116,832 | 0.66 | 779 | 1,030,819 | 0.76 | 0.86 (0.68-1.09) | 0.86 (0.68-1.09) | 0.85 (0.67-1.09) |
| 0–2 years | 144 | 215,273 | 0.67 | 1451 | 1,894,426 | 0.77 | 0.84 (0.71-1.00) | 0.84 (0.71-1.00) | 0.83 (0.69-1.00) |
| 0–3 years | 201 | 298,234 | 0.67 | 1999 | 2,610,658 | 0.77 | 0.87 (0.75-1.00) | 0.87 (0.75-1.00) | 0.87 (0.75-1.02) |
| 0–4 years | 256 | 367,661 | 0.70 | 2472 | 3,199,166 | 0.77 | 0.88 (0.78-1.01) | 0.89 (0.78-1.01) | 0.90 (0.78-1.03) |
| 0–5 years | 290 | 425,118 | 0.68 | 2895 | 3,676,485 | 0.79 | 0.85 (0.76-0.97) | 0.86 (0.76-0.97) | 0.87 (0.76-0.99) |
| **Denmark** | | | | | | | | | |
| **Time since start of follow-up** | **Bereaved cohort (N=335,618)** | | | **Matched comparators (N=3,112,812)** | | | **Unadjusted HR (95% CI)^a^** | **Adjusted HR (95% CI)^b^** | **Fully adjusted HR (95% CI)^c^** |
|  | **Number of events** | **Person-years at risk** | **Rate per 1,000** | **Number of events** | **Person-years at risk** | **Rate per 1,000** |  |  |  |
| Entire follow-up period | 1,633 | 2,480,208 | 0.66 | 15,143 | 20,598,508 | 0.74 | 0.89 (0.85-0.94) | 0.89 (0.85-0.94) | 0.89 (0.84-0.94) |
| 0–1 year | 195 | 319,695 | 0.61 | 1,823 | 2,941,548 | 0.62 | 0.98 (0.85-1.14) | 0.98 (0.85-1.14) | 0.96 (0.82-1.13) |
| 0–2 years | 378 | 610,623 | 0.62 | 3,591 | 5,560,006 | 0.65 | 0.97 (0.87-1.08) | 0.97 (0.87-1.08) | 0.96 (0.85-1.07) |
| 0–3 years | 522 | 874,934 | 0.60 | 5,219 | 7,882,814 | 0.66 | 0.91 (0.83-0.99) | 0.91 (0.83-1.00) | 0.90 (0.82-1.00) |
| 0–4 years | 669 | 1,113,647 | 0.60 | 6,582 | 9,930,864 | 0.66 | 0.91 (0.84-0.99) | 0.91 (0.84-0.99) | 0.90 (0.83-0.98) |
| 0–5 years | 792 | 1,327,997 | 0.60 | 7,864 | 11,729,931 | 0.67 | 0.89 (0.83-0.96) | 0.89 (0.83-0.96) | 0.88 (0.82-0.96) |

Abbreviations: HR, hazard ratio; CI, confidence interval

^a^Computed using Cox regression stratified by matched set to account for the matching variables of age, sex, county of residence (in Denmark) and general practice (in the UK).

^b^Adjusted for Charlson Comorbidity Index score.

^c^Complete-case analysis was used to handle missing data in the fully adjusted model. Notably, the number of events, person-years at risk and rate per 1,000 person-years in bereaved and matched comparators cohorts presented in this table were calculated for the full cohort in the unadjusted and adjusted models only. Adjusted additionally for smoking status, body mass index, alcohol consumption and deprivation status in the UK. The total number of bereaved and comparison persons was 115,167 and 937,661, respectively, after excluding those with missing values for body mass index, alcohol consumption and smoking status. Adjusted additionally for education duration in Denmark. The total number of bereaved and comparison persons was 291,859 and 2,684,403, respectively, after excluding those with missing values for education duration.

# **Supplementary material table 8. Association between partner bereavement and diagnosis of incident melanoma, sensitivity analysis restricted to patients eligible for linkage to Hospital Episode Statistics/Office for National Statistics death registration data.**

| **Time since start of follow-up** | **Bereaved cohort (N=100,994)** | | | **Matched comparators (N=945,400)** | | | **Unadjusted HR (95% CI)^a^** | **Adjusted HR (95% CI)^b^** | **Fully adjusted HR (95% CI)^c^** |
| --- | --- | --- | --- | --- | --- | --- | --- | --- | --- |
|  | **Number of events** | **Person-years at risk** | **Rate per 1,000** | **Number of events** | **Person-years at risk** | **Rate per 1,000** |  |  |  |
| Entire follow-up period | 392 | 532,240 | 0.74 | 3911 | 4,778,642 | 0.82 | 0.89 (0.80-0.99) | 0.89 (0.80-0.99) | 0.91 (0.81-1.02) |
| 0–1 year | 65 | 92,408 | 0.70 | 646 | 874,220 | 0.74 | 0.95 (0.73-1.22) | 0.94 (0.73-1.22) | 0.95 (0.73-1.24) |
| 0–2 years | 121 | 171,248 | 0.71 | 1237 | 1,617,879 | 0.76 | 0.89 (0.74-1.08) | 0.89 (0.74-1.08) | 0.91 (0.75-1.11) |
| 0–3 years | 161 | 238,803 | 0.67 | 1723 | 2,246,957 | 0.77 | 0.86 (0.73-1.02) | 0.86 (0.73-1.02) | 0.89 (0.75-1.06) |
| 0–4 years | 210 | 296,426 | 0.71 | 2145 | 2,775,635 | 0.77 | 0.91 (0.79-1.05) | 0.91 (0.79-1.05) | 0.94 (0.81-1.10) |
| 0–5 years | 242 | 345,271 | 0.70 | 2563 | 3,215,457 | 0.80 | 0.87 (0.76-1.00) | 0.87 (0.76-1.00) | 0.91 (0.79-1.04) |

Abbreviations: HR, hazard ratio; CI, confidence interval

^a^Computed using Cox regression stratified by matched set to account for the matching variables of age, sex, and general practice.

^b^Adjusted for Charlson Comorbidity Index score.

^c^Complete-case analysis was used to handle missing data in the fully adjusted model. Notably, the number of events, person-years at risk and rate per 1,000 person-years in the bereaved and matched comparators cohorts presented in this table were calculated for the full cohort in the unadjusted and adjusted models only. Adjusted additionally for smoking status, body mass index, alcohol consumption and deprivation status. The total number of bereaved and comparison persons was 89,189 and 769,863, respectively, after excluding those with missing values for body mass index, alcohol consumption and smoking status.

# **Supplementary material table 9. Association between partner bereavement and incident melanoma, *post-hoc* intention-to-treat analysis.**

| **UK** | | | | | | | | | |
| --- | --- | --- | --- | --- | --- | --- | --- | --- | --- |
| **Time since start of follow-up** | **Bereaved cohort (N=170,002)** | | | **Matched comparators (N=1,599,260)** | | | **Unadjusted HR (95% CI)^a^** | **Adjusted HR (95% CI)^b^** | **Fully adjusted HR (95% CI)^c^** |
|  | **Number of events** | **Person-years at risk** | **Rate per 1,000** | **Number of events** | **Person-years at risk** | **Rate per 1,000** |  |  |  |
| Entire follow-up period | 620 | 905,281 | 0.68 | 7532 | 9,459,567 | 0.80 | 0.86 (0.79-0.94) | 0.86 (0.79-0.94) | 0.87 (0.80-0.95) |
| 0–1 year | 104 | 155,818 | 0.67 | 1076 | 1,507,005 | 0.71 | 0.92 (0.75-1.13) | 0.92 (0.75-1.12) | 0.92 (0.74-1.14) |
| 0–2 years | 191 | 288,900 | 0.66 | 2083 | 2,837,199 | 0.73 | 0.87 (0.75-1.01) | 0.87 (0.75-1.01) | 0.88 (0.75-1.03) |
| 0–3 years | 260 | 402,950 | 0.65 | 2935 | 4,002,267 | 0.73 | 0.86 (0.76-0.98) | 0.86 (0.76-0.98) | 0.88 (0.77-1.01) |
| 0–4 years | 335 | 500,318 | 0.67 | 3751 | 5,016,177 | 0.75 | 0.89 (0.79-0.99) | 0.89 (0.79-0.99) | 0.91 (0.81-1.02) |
| 0–5 years | 382 | 582,924 | 0.66 | 4515 | 5,889,450 | 0.77 | 0.85 (0.76-0.94) | 0.85 (0.76-0.94) | 0.87 (0.78-0.97) |
| **Denmark** | | | | | | | | | |
| **Time since start of follow-up** | **Bereaved cohort (N=345,915)** | | | **Matched comparators (N=3,319,788)** | | | **Unadjusted HR (95% CI)^a^** | **Adjusted HR (95% CI)^b^** | **Fully adjusted HR (95% CI)^c^** |
|  | **Number of events** | **Person-years at risk** | **Rate per 1,000** | **Number of events** | **Person-years at risk** | **Rate per 1,000** |  |  |  |
| Entire follow-up period | 1,667 | 2,552,711 | 0.65 | 19,087 | 25,854,315 | 0.74 | 0.90 (0.86-0.95) | 0.90 (0.86-0.95) | 0.90 (0.85-0.95) |
| 0–1 year | 203 | 329,407 | 0.62 | 1,987 | 3,190,612 | 0.62 | 0.99 (0.86-1.15) | 0.99 (0.86-1.15) | 0.97 (0.83-1.13) |
| 0–2 years | 391 | 629,025 | 0.62 | 3,980 | 6,127,710 | 0.65 | 0.97 (0.87-1.08) | 0.97 (0.87-1.08) | 0.96 (0.85-1.07) |
| 0–3 years | 537 | 901,143 | 0.60 | 5,877 | 8,820,194 | 0.67 | 0.90 (0.83-0.99) | 0.90 (0.83-0.99) | 0.90 (0.82-0.99) |
| 0–4 years | 686 | 1,146,833 | 0.60 | 7,509 | 11,270,569 | 0.67 | 0.91 (0.84-0.98) | 0.91 (0.84-0.98) | 0.90 (0.83-0.98) |
| 0–5 years | 811 | 1,367,420 | 0.59 | 9,064 | 13,489,967 | 0.67 | 0.89 (0.83-0.96) | 0.89 (0.83-0.96) | 0.88 (0.82-0.95) |

Abbreviations: HR, hazard ratio; CI, confidence interval

^a^Computed using Cox regression stratified by matched set to account for the matching variables of age, sex, county of residence (in Denmark) and general practice (in the UK).

^b^Adjusted for Charlson Comorbidity Index score.

^c^Complete-case analysis was used to handle missing data in the fully adjusted model. Notably, the number of events, person-years at risk and rate per 1,000 person-years in the bereaved and matched comparators cohorts presented in this table were calculated for the full cohort in the unadjusted and adjusted models only. Adjusted additionally for smoking status, body mass index, alcohol consumption and deprivation status in the UK. The total number of bereaved and comparison persons was 150,547 and 1,306,385, respectively, after excluding those with missing values for body mass index, alcohol consumption and smoking status. Additionally adjusted for education duration in Denmark. The total number of bereaved and comparison persons was 300,686 and 2,857,617, respectively, after excluding those with missing values for education duration.

# **Supplementary material table 10. Association between partner bereavement and diagnosis of incident melanoma, *post-hoc* sensitivity analysis redefining the cohort using matching without replacement in the UK.**

| **Time since start of follow-up** | **Bereaved cohort (N=165,045)** | | | **Matched comparators (N=165,045)** | | | **Unadjusted HR (95% CI)^a^** | **Adjusted HR (95% CI)^b^** | **Fully adjusted HR (95% CI)^c^** |
| --- | --- | --- | --- | --- | --- | --- | --- | --- | --- |
|  | **Number of events** | **Person-years at risk** | **Rate per 1,000** | **Number of events** | **Person-years at risk** | **Rate per 1,000** |  |  |  |
| Entire follow-up period | 608 | 890,242 | 0.68 | 623 | 827,906 | 0.75 | 0.83 (0.73-0.95) | 0.83 (0.73-0.95) | 0.88 (0.76-1.02) |
| 0–1 year | 99 | 151,702 | 0.65 | 103 | 152,362 | 0.68 | 0.93 (0.70-1.23) | 0.93 (0.70-1.24) | 0.94 (0.68-1.30) |
| 0–2 years | 182 | 281,761 | 0.65 | 199 | 281,500 | 0.71 | 0.86 (0.70-1.06) | 0.86 (0.70-1.06) | 0.90 (0.71-1.15) |
| 0–3 years | 250 | 393,576 | 0.64 | 291 | 390,097 | 0.75 | 0.82 (0.69-0.99) | 0.82 (0.69-0.99) | 0.86 (0.70-1.06) |
| 0–4 years | 323 | 489,297 | 0.66 | 354 | 481,038 | 0.74 | 0.84 (0.71-0.99) | 0.84 (0.71-0.99) | 0.89 (0.74-1.08) |
| 0–5 years | 370 | 570,729 | 0.65 | 410 | 556,548 | 0.74 | 0.81 (0.70-0.95) | 0.81 (0.69-0.95) | 0.86 (0.72-1.03) |

Abbreviations: HR, hazard ratio; CI, confidence interval

^a^Computed using Cox regression stratified by matched set to account for the matching variables of age, sex, and general practice.

^b^Adjusted for Charlson Comorbidity Index score.

^c^Complete-case analysis was used to handle missing data in the fully adjusted model. Notably, the number of events, person-years at risk and rate per 1,000 person-years in the bereaved and matched comparators cohorts presented in this table were calculated for the full cohort in the unadjusted and adjusted models only. Adjusted additionally for smoking status, body mass index, alcohol consumption and deprivation status in the UK. The total number of bereaved and comparison persons was 133,813 and 133,813, respectively, after excluding those with missing values for body mass index, alcohol consumption and smoking status.

# **Supplementary material table 11. Association between partner bereavement and diagnosis of incident melanoma, sensitivity analysis censoring at end of partnership.**

| **Time since start of follow-up** | **Bereaved cohort (N=345,915)** | | | **Matched comparators (N=3,319,788)** | | | **Unadjusted HR (95% CI)^a^** | **Adjusted HR (95% CI)^b^** | **Fully adjusted HR (95% CI)^c^** |
| --- | --- | --- | --- | --- | --- | --- | --- | --- | --- |
|  | **Number of events** | **Person-years at risk** | **Rate per 1,000** | **Number of events** | **Person-years at risk** | **Rate per 1,000** |  |  |  |
| Entire follow-up period | 1,667 | 2,552,711 | 0.65 | 16,031 | 21,752,371 | 0.74 | 0.88 (0.84-0.93) | 0.89 (0.84-0.93) | 0.89 (0.84-0.94) |
| 0–1 year | 203 | 329,407 | 0.62 | 1,943 | 3,135,759 | 0.62 | 0.99 (0.86-1.15) | 0.99 (0.86-1.15) | 0.97 (0.83-1.13) |
| 0–2 years | 391 | 629,025 | 0.62 | 3,823 | 5,923,013 | 0.65 | 0.98 (0.88-1.08) | 0.98 (0.88-1.08) | 0.96 (0.86-1.08) |
| 0–3 years | 537 | 901,143 | 0.60 | 5,561 | 8,391,539 | 0.66 | 0.91 (0.83-0.99) | 0.91 (0.83-0.99) | 0.90 (0.82-0.99) |
| 0–4 years | 686 | 1,146,833 | 0.60 | 7,013 | 10,563,940 | 0.66 | 0.91 (0.84-0.98) | 0.91 (0.84-0.98) | 0.90 (0.83-0.98) |
| 0–5 years | 811 | 1,367,420 | 0.59 | 8,383 | 12,468,168 | 0.67 | 0.88 (0.82-0.95) | 0.89 (0.82-0.95) | 0.88 (0.81-0.95) |

Abbreviations: HR, hazard ratio; CI, confidence interval

^a^Computed using Cox regression stratified by matched set to account for the matching variables of age, sex, county of residence.

^b^Adjusted for Charlson Comorbidity Index score.

^c^Complete-case analysis was used to handle missing data in the fully adjusted model. Notably, the number of events, person-years at risk and rate per 1,000 person-years in the bereaved and matched comparators cohorts presented in this table were calculated for the full cohort in the unadjusted and adjusted models only. Adjusted additionally for education duration. The total number of bereaved and comparison persons was 300,686 and 2,857,617, respectively, after excluding those with missing values for education duration.

# **Supplementary material table 12. Association between partner bereavement and diagnosis of incident melanoma, sensitivity analysis including only histologically verified diagnoses in the outcome definition.**

| **Time since start of follow-up** | **Bereaved cohort (N=345,915)** | | | **Matched comparators (N=3,319,788)** | | | **Unadjusted HR (95% CI)^a^** | **Adjusted HR (95% CI)^b^** | **Fully adjusted HR (95% CI)^c^** |
| --- | --- | --- | --- | --- | --- | --- | --- | --- | --- |
|  | **Number of events** | **Person-years at risk** | **Rate per 1,000** | **Number of events** | **Person-years at risk** | **Rate per 1,000** |  |  |  |
| Entire follow-up period | 1,663 | 2,552,711 | 0.65 | 16,143 | 22,027,622 | 0.73 | 0.88 (0.84-0.93) | 0.88 (0.84-0.93) | 0.88 (0.84-0.94) |
| 0–1 year | 202 | 329,407 | 0.61 | 1,942 | 3,137,524 | 0.62 | 0.99 (0.85-1.14) | 0.99 (0.85-1.14) | 0.97 (0.83-1.13) |
| 0–2 years | 390 | 629,025 | 0.62 | 3,821 | 5,931,262 | 0.64 | 0.98 (0.88-1.08) | 0.98 (0.88-1.08) | 0.96 (0.86-1.08) |
| 0–3 years | 536 | 901,143 | 0.59 | 5,560 | 8,411,041 | 0.66 | 0.91 (0.83-0.99) | 0.91 (0.83-0.99) | 0.90 (0.82-0.99) |
| 0–4 years | 683 | 1,146,833 | 0.60 | 7,013 | 10,598,701 | 0.66 | 0.90 (0.84-0.98) | 0.90 (0.84-0.98) | 0.90 (0.82-0.98) |
| 0–5 years | 808 | 1,367,420 | 0.59 | 8,386 | 12,521,393 | 0.67 | 0.88 (0.82-0.95) | 0.88 (0.82-0.95) | 0.88 (0.81-0.95) |

Abbreviations: HR, hazard ratio; CI, confidence interval

^a^Computed using Cox regression stratified by matched set to account for the matching variables of age, sex, county of residence.

^b^Adjusted for Charlson Comorbidity Index score.

^c^Complete-case analysis was used to handle missing data in the fully adjusted model. Notably, the number of events, person-years at risk and rate per 1,000 person-years in the bereaved and matched comparators cohorts presented in this table were calculated for the full cohort in the unadjusted and adjusted models only. Adjusted additionally for education duration. The total number of bereaved and comparison persons was 300,686 and 2,857,617, respectively, after excluding those with missing values for education duration.

# **Supplementary material table 13. Association between partner bereavement and melanoma mortality in patients with melanoma, overall and by time since melanoma diagnosis.**

| **Time since melanoma diagnosis** | **Unexposed time** | | **Exposed time** | | **Unadjusted HR (95% CI)** | **Adjusted HR^a^**  **(95% CI)** | **Fully adjusted HR^b^ (95% CI)** |
| --- | --- | --- | --- | --- | --- | --- | --- |
|  | **Number of deaths** | **Person-years at risk** | **Number of deaths** | **Person-years at risk** |  |  |  |
| **UK** | | | | | | | |
| Entire follow-up period | 304 | 15,923 | 47 | 1701 | 1.60 (1.17-2.17) | 1.30 (0.93-1.82) | 1.28 (0.88-1.86) |
| 0–1 year | 89 | 3019 | 10 | 262 | 1.29 (0.67-2.49) | 1.00 (0.50-2.00) | 1.03 (0.49-2.17) |
| 0–2 years | 166 | 5521 | 21 | 504 | 1.38 (0.88-2.18) | 1.01 (0.62-1.63) | 0.93 (0.53-1.63) |
| 0–3 years | 214 | 7612 | 32 | 698 | 1.63 (1.12-2.36) | 1.23 (0.82-1.83) | 1.21 (0.77-1.90) |
| 0–4 years | 241 | 9341 | 38 | 861 | 1.71 (1.22-2.41) | 1.35 (0.93-1.96) | 1.30 (0.86-1.97) |
| 0–5 years | 266 | 10,772 | 40 | 1003 | 1.62 (1.16-2.26) | 1.30 (0.91-1.86) | 1.25 (0.84-1.87) |
| **Denmark** | | | | | | | |
| Entire follow-up period | 2,254 | 138,425 | 446 | 15,764 | 1.89 (1.70-2.09) | 1.16 (1.04-1.30) | 1.14 (1.01-1.29) |
| 0–1 year | 570 | 21,280 | 132 | 2,062 | 2.39 (1.98-2.89) | 1.34 (1.08-1.65) | 1.22 (0.96-1.56) |
| 0–2 years | 1,047 | 39,803 | 224 | 3,910 | 2.18 (1.89-2.52) | 1.21 (1.03-1.41) | 1.16 (0.96-1.38) |
| 0–3 years | 1,411 | 55,974 | 293 | 5,561 | 2.09 (1.85-2.37) | 1.21 (1.05-1.39) | 1.16 (1.00-1.36) |
| 0–4 years | 1,661 | 69,992 | 330 | 7,020 | 1.99 (1.77-2.24) | 1.19 (1.04-1.35) | 1.16 (1.00-1.34) |
| 0–5 years | 1,850 | 82,098 | 367 | 8,317 | 1.97 (1.76-2.21) | 1.19 (1.05-1.35) | 1.16 (1.02-1.34) |

Abbreviations: HR, hazard ratio; CI, confidence interval

^a^Adjusted for age, gender and Charlson Comorbidity Index score (excluded all codes indicating skin cancers).

^b^Complete-case analysis was used to handle missing data in the fully adjusted model. Notably, the number of events, person-years at risk and rate per 1,000 person-years in the bereaved and matched comparators cohorts presented in this table were calculated for the full cohort in the unadjusted and adjusted models only. Additionally adjusted for deprivation status, smoking status, body mass index, alcohol consumption in the UK. The total number of patients was 3233 after excluding those with missing values for body mass index, alcohol consumption and smoking status. Additionally adjusted for education duration in Denmark. The total number of patients was 23,882 after excluding those with missing values for education duration.

# **Supplementary material table 14. Association between partner bereavement and mortality in patients with melanoma. Unadjusted and adjusted hazard ratios for the full cohort and the complete case cohort.**

| **Time since start of follow-up** | **Unadjusted HR (95% CI)^a^** | | **Adjusted HR (95% CI)^a^** | |
| --- | --- | --- | --- | --- |
|  | **Full cohort (N=3597)** | **Complete case cohort (N=3233)** | **Full cohort (N=3597)** | **Complete case cohort (N=3233)** |
| **UK** | | | | |
| Entire follow-up period | 1.60 (1.17-2.17) | 1.68 (1.19-2.37) | 1.30 (0.93-1.82) | 1.31 (0.90-1.89) |
| 0–1 year | 1.29 (0.67-2.49) | 1.60 (0.80-3.21) | 1.00 (0.50-2.00) | 1.10 (0.53-2.30) |
| 0–2 years | 1.38 (0.88-2.18) | 1.39 (0.83-2.34) | 1.01 (0.62-1.63) | 0.98 (0.57-1.71) |
| 0–3 years | 1.63 (1.12-2.36) | 1.73 (1.14-2.61) | 1.23 (0.82-1.83) | 1.25 (0.80-1.95) |
| 0–4 years | 1.71 (1.22-2.41) | 1.80 (1.23-2.63) | 1.35 (0.93-1.96) | 1.34 (0.89-2.02) |
| 0–5 years | 1.62 (1.16-2.26) | 1.71 (1.19-2.47) | 1.30 (0.91-1.86) | 1.30 (0.88-1.93) |
|  | **Unadjusted HR (95% CI)^a^** | | **Adjusted HR (95% CI)^a^** | |
| **Time since start of follow-up** | **Full cohort (N=24,911)** | **Complete case cohort (N=23,882)** | **Full cohort (N=24,911)** | **Complete case cohort (N=23,882)** |
| **Denmark** | | | | |
| Entire follow-up period | 1.89 (1.70-2.09) | 1.78 (1.59-1.99) | 1.16 (1.04-1.30) | 1.17 (1.03-1.32) |
| 0–1 year | 2.39 (1.98-2.89) | 2.06 (1.65-2.56) | 1.34 (1.08-1.65) | 1.25 (0.98-1.59) |
| 0–2 years | 2.18 (1.89-2.52) | 1.97 (1.67-2.32) | 1.21 (1.03-1.41) | 1.18 (0.99-1.42) |
| 0–3 years | 2.09 (1.85-2.37) | 1.92 (1.67-2.21) | 1.21 (1.05-1.39) | 1.19 (1.02-1.39) |
| 0–4 years | 1.99 (1.77-2.24) | 1.86 (1.63-2.12) | 1.19 (1.04-1.35) | 1.19 (1.03-1.37) |
| 0–5 years | 1.97 (1.76-2.21) | 1.84 (1.63-2.09) | 1.19 (1.05-1.35) | 1.19 (1.04-1.37) |

Abbreviation: HR, hazard ratio; CI, confidence interval

^a^Adjusted for age, sex and Charlson Comorbidity Index score.

# **Supplementary material table 15. Association between partner bereavement and all-cause mortality in patients with melanoma, overall and by time since melanoma diagnosis, *post hoc.***

| **Time intervals following melanoma diagnosis** | **Unexposed time** | | **Exposed time** | | **Unadjusted HR**  **(95% CI)** | **Adjusted HR^a^**  **(95% CI)** | **Fully adjusted HR^b^**  **(95% CI)** |
| --- | --- | --- | --- | --- | --- | --- | --- |
|  | **Number of deaths** | **Person-years at risk** | **Number of deaths** | **Person-years at risk** |  |  |  |
| **UK** | | | | | | | |
| Entire follow-up period | 597 | 15,923 | 135 | 1701 | 2.25 (1.86-2.71) | 1.31 (1.07-1.61) | 1.23 (0.98-1.54) |
| 0–1 year | 169 | 3019 | 27 | 262 | 1.86 (1.24-2.79) | 1.05 (0.68-1.62) | 1.00 (0.63-1.61) |
| 0–2 years | 286 | 5521 | 50 | 504 | 1.93 (1.43-2.61) | 1.07 (0.78-1.48) | 1.00 (0.70-1.44) |
| 0–3 years | 369 | 7612 | 73 | 698 | 2.17 (1.69-2.79) | 1.24 (0.94-1.62) | 1.16 (0.86-1.58) |
| 0–4 years | 423 | 9341 | 87 | 861 | 2.25 (1.78-2.83) | 1.32 (1.03-1.70) | 1.23 (0.93-1.62) |
| 0–5 years | 476 | 10,772 | 95 | 1003 | 2.16 (1.74-2.70) | 1.27 (1.00-1.61) | 1.18 (0.90-1.53) |
| **Denmark** |  |  |  |  |  |  |  |
| Entire follow-up period | 3,774 | 138,425 | 1,201 | 15,764 | 2.92 (2.73-3.11) | 1.29 (1.20-1.38) | 1.26 (1.16-1.36) |
| 0–1 year | 786 | 21,280 | 211 | 2,062 | 2.77 (2.38-3.22) | 1.30 (1.10-1.54) | 1.22 (1.00-1.48) |
| 0–2 years | 1,471 | 39,803 | 390 | 3,910 | 2.70 (2.42-3.02) | 1.22 (1.07-1.38) | 1.17 (1.02-1.35) |
| 0–3 years | 2,017 | 55,974 | 540 | 5,561 | 2.70 (2.45-2.97) | 1.23 (1.11-1.37) | 1.18 (1.04-1.33) |
| 0–4 years | 2,432 | 69,992 | 652 | 7,020 | 2.68 (2.46-2.92) | 1.23 (1.11-1.35) | 1.19 (1.07-1.33) |
| 0–5 years | 2,759 | 82,098 | 754 | 8,317 | 2.71 (2.50-2.94) | 1.24 (1.13-1.35) | 1.20 (1.09-1.33) |

Abbreviations: HR, hazard ratio; CI, confidence interval

^a^Adjusted for age, gender and Charlson Comorbidity Index

^b^Complete-case analysis was used to handle missing data in the fully adjusted model. Notably, the number of events, person-years at risk and rate per 1,000 person-years in the bereaved and matched comparators cohorts presented in this table were calculated for the full cohort in the unadjusted and adjusted models only. Additionally adjusted for deprivation status, smoking status, body mass index, and alcohol consumption in the UK. The total number of persons was 3233 after excluding persons with missing values for body mass index, alcohol consumption and smoking status. Additionally adjusted for education duration in Denmark. The total number of patients was 23,882 after excluding those with missing values for education duration.

# **Supplementary material table 16. Association between partner bereavement and melanoma mortality in patients with melanoma, subgroup analysis by age and sex.**

|  | **Unexposed time** | | **Exposed time** | | **Unadjusted HR**  **(95% CI)** | **Adjusted HR^a^**  **(95% CI)** | **Interaction p-value** | **Fully adjusted HR^b^ (95% CI)** |
| --- | --- | --- | --- | --- | --- | --- | --- | --- |
|  | **Number of deaths** | **Person-years at risk** | **Number of deaths** | **Person-years at risk** |  |  |  |  |
| **UK** | | | | | | | | |
| *Age* | | | | | | | | |
| <50 | ^c^ | 2058 | ^c^ | 30 | NA | NA | p=0.02 | NA |
| 50–59 | ^c^ | 4667 | ^c^ | 143 | NA | NA |  | NA |
| 60–69 | 91 | 4981 | 8 | 383 | 1.53 (0.74-3.16) | 1.81 (0.87-3.75) |  | 2.28 (1.04-5.00) |
| 70–79 | 98 | 3355 | 13 | 662 | 0.81 (0.46-1.45) | 0.87 (0.49-1.55) |  | 0.77 (0.40-1.48) |
| 80+ | 31 | 863 | 26 | 483 | 1.62 (0.96-2.74) | 1.87 (1.11-3.16) |  | 1.82 (1.01-3.28) |
| *Sex* | | | | | | | | |
| Female | 92 | 7541 | 26 | 1053 | 2.25 (1.45-3.48) | 1.75 (1.11-2.76) | p=0.09 | 1.56 (0.92-2.66) |
| Male | 212 | 8382 | 21 | 648 | 1.38 (0.88-2.15) | 1.01 (0.64-1.61) |  | 1.11 (0.67-1.81) |
| **Denmark** | | | | | | | | |
| *Age* | | | | | | | | |
| <50 | 459 | 59,569 | 9 | 940 | 1.55 (0.80-3.00) | 1.59 (0.82-3.08) |  | 1.60 (0.82-3.10) |
| 50–59 | 459 | 33,595 | 19 | 2,110 | 0.84 (0.53-1.34) | 0.96 (0.61-1.53) |  | 0.91 (0.57-1.47) |
| 60–69 | 595 | 28,261 | 72 | 4,537 | 0.93 (0.73-1.19) | 1.03 (0.80-1.32) | p=0.56 | 1.03 (0.80-1.33) |
| 70–79 | 499 | 13,477 | 151 | 5,094 | 0.97 (0.81-1.17) | 1.16 (0.96-1.40) |  | 1.15 (0.94-1.39) |
| 80+ | 242 | 3,523 | 195 | 3,083 | 1.02 (0.85-1.24) | 1.17 (0.95-1.44) |  | 1.16 (0.89-1.52) |
| *Sex* | | | | | | | | |
| Female | 711 | 75,333 | 241 | 10,742 | 2.58 (2.23-2.98) | 1.11 (0.93-1.33) |  | 1.09 (0.90-1.31) |
| Male | 1,543 | 63,092 | 205 | 5,022 | 1.78 (1.53-2.05) | 1.11 (0.96-1.30) | p=0.11 | 1.10 (0.93-1.31) |

Abbreviations: NA, not applicable; HR, hazard ratio; CI, confidence interval

^a^Adjusted for gender and Charlson Comorbidity Index score in subgroup analysis according to age. Adjusted for age and Charlson Comorbidity Index score in subgroup analysis according to gender.

^b^Complete-case analysis was used to handle missing data in the fully adjusted model. Notably, the number of events, person-years at risk and rate per 1,000 person-years in the bereaved and matched comparators cohorts presented in this table were calculated for the full cohort in the unadjusted and adjusted models only. Additionally adjusted for deprivation status, smoking status, body mass index and alcohol consumption in the UK. Additionally adjusted for education duration in Denmark. The total number of patients was 23,882 after excluding those with missing values for education duration.

^c^If there were fewer than 5 patients the exact number was withheld in accordance with the confidentiality rules of the CPRD/Danish registries

# **Supplementary material table 17. Association between partner bereavement and melanoma-specific mortality in patients with melanoma, subgroup analysis by cancer stage at diagnosis in Denmark.**

| **Melanoma mortality (N=20,329)** | | | | | | | | |
| --- | --- | --- | --- | --- | --- | --- | --- | --- |
|  | **Unexposed time** | | **Exposed time** | | **Unadjusted HR**  **(95% CI)** | **Adjusted HR^a^**  **(95% CI)** | **Interaction p-value** | **Fully adjusted HR^b^ (95% CI)** |
|  | **Number of deaths** | **Person-years at risk** | **Number of deaths** | **Person-years at risk** |  |  |  |  |
| *Cancer stage at diagnosis* | | | | | | | | |
| Localized | 1,009 | 111,726 | 225 | 12,566 | 2.10 (1.82-2.43) | 1.12 (0.96-1.32) | p=0.076 | 1.07 (0.90-1.28) |
| Regional | 392 | 6,920 | 62 | 693 | 1.63 (1.25-2.14) | 1.06 (0.79-1.44) |  | 1.16 (0.84-1.59) |
| Distant | 161 | 522 | 16 | 73 | 0.95 (0.57-1.59) | 0.90 (0.52-1.55) |  | 0.82 (0.46-1.47) |

Abbreviations: HR, hazard ratio; CI, confidence interval

^a^Adjusted for age, gender and Charlson Comorbidity Index score.

^b^Complete-case analysis was used to handle missing data in the fully adjusted model. Notably, the number of events, person-years at risk and rate per 1,000 person-years in the bereaved and matched comparators cohorts presented in this table were calculated for the full cohort in the unadjusted and adjusted models only. Additionally adjusted for education duration. In Denmark the total number of patients was 19,529 after excluding those with missing values for education duration.

# **Supplementary material table 18. Characteristics of patients with melanoma in the UK (not limited to those with data linkage to the Office of National Statistics death registration).**

|  | **No. (%)** |
| --- | --- |
| *Total* | 9206 |
| *Age, years* |  |
| Range | 31.0-99.6 |
| Median (IQR) | 66.9 (58.3-75.3) |
| Age groups (years) |  |
| <50 | 669 (7.3) |
| 50–59 | 2058 (22.4) |
| 60–69 | 2780 (30.2) |
| 70–79 | 2454 (26.7) |
| 80+ | 1245 (13.5) |
| *Sex* |  |
| Women | 4308 (46.8) |
| Men | 4898 (53.2) |
| *Comorbidity burden^a^* |  |
| Low | 4951 (53.8) |
| Intermediate | 2869 (31.2) |
| High | 1386 (15.1) |
| *Index of multiple deprivation* |  |
| 1 (least deprived) | 2844 (30.9) |
| 2 | 2187 (23.8) |
| 3 | 2016 (21.9) |
| 4 | 1399 (15.2) |
| 5 (most deprived) | 760 (8.3) |
| *Follow-up (years)* |  |
| Total | 49,540 |
| Median (IQR) | 3.8 (1.6-7.5) |

Abbreviation: IQR, interquartile range

^a^Comorbidity burden was measured by the Charlson Comorbidity Index score. Comorbidity burden was defined on the date of melanoma diagnosis using the Charlson Comorbidity Index score, categorised as low (0 point), intermediate (1–2 points), and high (≥3 points).

# **Supplementary material table 19. Association between partner bereavement and all-cause mortality among patients with melanoma (not limited to those with data linkage to the Office for National Statistics death registration), *post hoc.***

| **Time intervals following melanoma diagnosis** | **Unexposed time** | | **Exposed time** | | **Unadjusted HR**  **(95% CI)** | **Adjusted HR^a^**  **(95% CI)** | **Fully adjusted HR^b^**  **(95% CI)** |
| --- | --- | --- | --- | --- | --- | --- | --- |
|  | **Number of deaths** | **Person-years at risk** | **Number of deaths** | **Person-years at risk** |  |  |  |
| Entire follow-up period | 1549 | 44,539 | 366 | 5001 | 2.23 (1.99-2.50) | 1.33 (1.17-1.50) | 1.31 (1.15-1.50) |
| 0–1 year | 399 | 7792 | 78 | 690 | 2.22 (1.74-2.82) | 1.28 (0.98-1.66) | 1.38 (1.04-1.82) |
| 0–2 years | 702 | 14,362 | 136 | 1306 | 2.14 (1.78-2.57) | 1.27 (1.04-1.54) | 1.26 (1.02-1.57) |
| 0–3 years | 927 | 19,904 | 175 | 1843 | 2.05 (1.74-2.41) | 1.23 (1.03-1.46) | 1.21 (1.00-1.46) |
| 0–4 years | 1075 | 24,571 | 204 | 2322 | 2.03 (1.75-2.36) | 1.23 (1.05-1.45) | 1.21 (1.02-1.44) |
| 0–5 years | 1190 | 28,540 | 226 | 2748 | 2.00 (1.74-2.31) | 1.20 (1.03-1.39) | 1.17 (0.99-1.38) |

^b^Complete-case analysis was used to handle missing data in the fully adjusted model. Notably, the number of events, person-years at risk and rate per 1,000 person-years in the bereaved and matched comparators cohorts presented in this table were calculated for the full cohort in the unadjusted and adjusted models only. Additionally adjusted for deprivation status, smoking status, body mass index, and alcohol consumption in the UK. The total number of persons was 8373 after excluding persons with missing values for body mass index, alcohol consumption and smoking status. Additionally adjusted for education duration in Denmark. The total number of patients was 23,882 after excluding persons with missing values for education duration.

# **Supplementary material table 20. Association between partner bereavement and melanoma mortality in melanoma patients, sensitivity analysis excluding those who experienced bereavement before or on the date of melanoma diagnosis.**

| **UK** | | | | | | | |
| --- | --- | --- | --- | --- | --- | --- | --- |
| **Melanoma mortality (N=3,326)** | | | | | | | |
| **Time intervals following melanoma diagnosis** | **Unexposed time** | | **Exposed time** | | **Unadjusted HR (95% CI)** | **Adjusted HR^a^**  **(95% CI)** | **Fully adjusted HR^b^ (95% CI)** |
|  | **Number of deaths** | **Person-years at risk** | **Number of deaths** | **Person-years at risk** |  |  |  |
| Entire follow-up period | 304 | 15,923 | 15 | 828 | 1.48 (0.88-2.51) | 1.34 (0.78-2.30) | 1.50 (0.84-2.71) |
| 0–1 year | ^c^ | 3019 | ^c^ | 22 | 3.09 (0.76-12.63) | 2.83 (0.68-11.81) | 2.90 (0.68-12.39) |
| 0–2 years | ^c^ | 5521 | ^c^ | 84 | 0.78 (0.19-3.15) | 0.70 (0.17-2.87) | 0.87 (0.21-3.62) |
| 0–3 years | 214 | 7612 | 6 | 155 | 1.42 (0.63-3.21) | 1.28 (0.56-2.94) | 1.61 (0.69-3.75) |
| 0–4 years | 241 | 9341 | 8 | 229 | 1.49 (0.73-3.03) | 1.38 (0.67-2.85) | 1.67 (0.80-3.50) |
| 0–5 years | 266 | 10,772 | 9 | 305 | 1.36 (0.70-2.65) | 1.25 (0.63-2.47) | 1.51 (0.75-3.03) |
| **Denmark** | | | | | | | |
| **Melanoma mortality (N=22,749)** | | | | | | | |
| Entire follow-up period | 2,254 | 138,425 | 121 | 6,771 | 1.82 (1.51-2.19) | 1.20 (0.99-1.46) | 1.20 (0.97-1.48) |
| 0–1 year | 570 | 21,280 | 10 | 112 | 3.31 (1.77-6.20) | 2.02 (1.07-3.80) | 2.15 (1.06-4.35) |
| 0–2 years | 1,047 | 39,803 | 25 | 410 | 2.36 (1.59-3.52) | 1.36 (0.91-2.03) | 1.47 (0.94-2.30) |
| 0–3 years | 1,411 | 55,974 | 41 | 812 | 2.09 (1.53-2.86) | 1.27 (0.93-1.75) | 1.31 (0.92-1.85) |
| 0–4 years | 1,661 | 69,992 | 57 | 1,273 | 2.05 (1.57-2.68) | 1.29 (0.98-1.69) | 1.31 (0.97-1.77) |
| 0–5 years | 1,850 | 82,098 | 75 | 1,780 | 2.10 (1.67-2.65) | 1.34 (1.06-1.70) | 1.35 (1.04-1.76) |

Abbreviations: HR, hazard ratio; CI, confidence interval

^a^Adjusted for Charlson Comorbidity Index score.

^b^Complete-case analysis was used to handle missing data in the fully adjusted model. Notably, the number of events, person-years at risk and rate per 1,000 person-years in the bereaved and matched comparators cohorts presented in this table were calculated for the full cohort in the unadjusted and adjusted models only. Adjusted additionally for smoking status, body mass index, alcohol consumption and deprivation status in the UK. The total number of patients was 2986 after excluding those with missing values for body mass index, alcohol consumption and smoking status. Additionally adjusted for education duration in Denmark. The total number of patients was 21,985 after excluding those with missing values for education duration.

^c^If there were fewer than 5 patients the exact number was withheld in accordance with the confidentiality rules of the CPRD/Danish registries

# **Supplementary material table 21. Association between partner bereavement and melanoma mortality, sensitivity analysis *post hoc* excluding patients who had lost their partner or were no longer in a partnership with their partner 3 years prior to melanoma diagnosis.**

| **Melanoma mortality (N=22,108)** | | | | | | | |
| --- | --- | --- | --- | --- | --- | --- | --- |
| **Time intervals following melanoma diagnosis** | **Unexposed time** | | **Exposed time** | | **Unadjusted HR (95% CI)** | **Adjusted HR^a^**  **(95% CI)** | **Fully adjusted HR^b^ (95% CI)** |
|  | **Number of deaths** | **Person-years at risk** | **Number of deaths** | **Person-years at risk** |  |  |  |
| Entire follow-up period | 2,159 | 132,939 | 224 | 9,976 | 1.81 (1.57-2.07) | 1.16 (1.00-1.34) | 1.15 (0.98-1.34) |
| 0–1 year | 538 | 20,103 | 43 | 712 | 2.25 (1.65-3.07) | 1.31 (0.95-1.81) | 1.13 (0.77-1.67) |
| 0–2 years | 998 | 37,646 | 83 | 1,509 | 2.08 (1.66-2.60) | 1.18 (0.93-1.49) | 1.11 (0.85-1.46) |
| 0–3 years | 1,343 | 53,014 | 111 | 2,328 | 1.91 (1.57-2.32) | 1.14 (0.93-1.40) | 1.08 (0.86-1.36) |
| 0–4 years | 1,581 | 66,381 | 139 | 3,136 | 1.92 (1.61-2.28) | 1.18 (0.98-1.41) | 1.15 (0.94-1.41) |
| 0–5 years | 1,767 | 77,960 | 166 | 3,922 | 1.95 (1.66-2.29) | 1.22 (1.03-1.44) | 1.20 (0.99-1.44) |

Abbreviations: HR, hazard ratio; CI, confidence interval

^a^Adjusted for Charlson Comorbidity Index score.

^b^Complete-case analysis was used to handle missing data in the fully adjusted model. Notably, the number of events, person-years at risk and rate per 1,000 person-years in the bereaved and matched comparators cohorts presented in this table were calculated for the full cohort in the unadjusted and adjusted models only. Additionally adjusted for education duration in Denmark. The total number of patients was 21,268 after excluding those with missing values for education duration.

# **Supplementary material table 22. Association between partner bereavement and melanoma mortality, sensitivity analysis censoring follow-up at the end of the partnership, and excluding persons if this occurred before melanoma diagnosis.**

| **Melanoma mortality (N=23,206)** | | | | | | | |
| --- | --- | --- | --- | --- | --- | --- | --- |
| **Time intervals following melanoma diagnosis** | **Unexposed time** | | **Exposed time** | | **Unadjusted HR (95% CI)** | **Adjusted HR^a^**  **(95% CI)** | **Fully adjusted HR^b^ (95% CI)** |
|  | **Number of deaths** | **Person-years at risk** | **Number of deaths** | **Person-years at risk** |  |  |  |
| Entire follow-up period | 2,114 | 126,172 | 438 | 15,245 | 1.87 (1.69-2.07) | 1.17 (1.04-1.31) | 1.14 (1.01-1.29) |
| 0–1 year | 536 | 19,715 | 130 | 1,997 | 2.39 (1.98-2.90) | 1.35 (1.09-1.68) | 1.23 (0.96-1.57) |
| 0–2 years | 989 | 36,817 | 220 | 3,789 | 2.16 (1.87-2.50) | 1.21 (1.03-1.43) | 1.16 (0.96-1.39) |
| 0–3 years | 1,328 | 51,694 | 287 | 5,395 | 2.08 (1.83-2.36) | 1.21 (1.05-1.40) | 1.16 (0.99-1.36) |
| 0–4 years | 1,562 | 64,546 | 323 | 6,811 | 1.97 (1.75-2.22) | 1.19 (1.04-1.36) | 1.16 (1.00-1.34) |
| 0–5 years | 1,740 | 75,589 | 359 | 8,066 | 1.95 (1.74-2.18) | 1.19 (1.05-1.35) | 1.16 (1.01-1.33) |

Abbreviations: HR, hazard ratio; CI, confidence interval

^a^Adjusted for Charlson Comorbidity Index score.

^b^Complete-case analysis was used to handle missing data in the fully adjusted model. Notably, the number of events, person-years at risk and rate per 1,000 person-years in the bereaved and matched comparators cohorts presented in this table were calculated for the full cohort in the unadjusted and adjusted models only. Additionally adjusted for education duration in Denmark. The total number of patients was 22,196 after excluding those with missing values for education duration.

# **Supplementary material table 23. Association between partner bereavement and melanoma mortality, sensitivity analysis *post hoc* censoring follow-up at emigration or end of the partnership, and excluding persons if either event occurred before melanoma diagnosis.**

| **Melanoma mortality (N=23,170)** | | | | | | | |
| --- | --- | --- | --- | --- | --- | --- | --- |
| **Time intervals following melanoma diagnosis** | **Unexposed time** | | **Exposed time** | | **Unadjusted HR (95% CI)** | **Adjusted HR^a^**  **(95% CI)** | **Fully adjusted HR^b^ (95% CI)** |
|  | **Number of deaths** | **Person-years at risk** | **Number of deaths** | **Person-years at risk** |  |  |  |
| Entire follow-up period | 2,109 | 125,898 | 438 | 15,245 | 1.87 (1.69-2.07) | 1.16 (1.04-1.31) | 1.14 (1.01-1.29) |
| 0–1 year | 535 | 19,679 | 130 | 1,997 | 2.39 (1.98-2.90) | 1.35 (1.09-1.68) | 1.22 (0.96-1.56) |
| 0–2 years | 987 | 36,746 | 220 | 3,789 | 2.16 (1.87-2.50) | 1.21 (1.03-1.42) | 1.15 (0.96-1.39) |
| 0–3 years | 1,326 | 51,589 | 287 | 5,395 | 2.07 (1.83-2.36) | 1.21 (1.05-1.40) | 1.16 (0.99-1.36) |
| 0–4 years | 1,559 | 64,410 | 323 | 6,811 | 1.97 (1.75-2.22) | 1.19 (1.04-1.35) | 1.15 (1.00-1.34) |
| 0–5 years | 1,737 | 75,427 | 359 | 8,066 | 1.95 (1.74-2.18) | 1.19 (1.05-1.35) | 1.16 (1.01-1.33) |

Abbreviations: HR, hazard ratio; CI, confidence interval

^a^Adjusted for Charlson Comorbidity Index score.

^b^Complete-case analysis was used to handle missing data in the fully adjusted model. Notably, the number of events, person-years at risk and rate per 1,000 person-years in the bereaved and matched comparators cohorts presented in this table were calculated for the full cohort in the unadjusted and adjusted models only. Additionally adjusted for education duration in Denmark. The total number of patients was 22,163 after excluding those with missing values for education duration.

# **Supplementary material table 24. Association between partner bereavement and melanoma mortality, sensitivity analysis including only histologically verified diagnoses in the outcome definition.**

| **Melanoma mortality (N=24,878)** | | | | | | | |
| --- | --- | --- | --- | --- | --- | --- | --- |
| **Time intervals following melanoma diagnosis** | **Unexposed time** | | **Exposed time** | | **Unadjusted HR (95% CI)** | **Adjusted HR^a^**  **(95% CI)** | **Fully adjusted HR^b^ (95% CI)** |
|  | **Number of deaths** | **Person-years at risk** | **Number of deaths** | **Person-years at risk** |  |  |  |
| Entire follow-up period | 2,251 | 138,293 | 445 | 15,724 | 1.89 (1.70-2.09) | 1.16 (1.04-1.30) | 1.15 (1.01-1.30) |
| 0–1 year | 567 | 21,258 | 131 | 2,057 | 2.39 (1.97-2.89) | 1.34 (1.09-1.66) | 1.23 (0.97-1.57) |
| 0–2 years | 1,044 | 39,761 | 223 | 3,901 | 2.18 (1.88-2.52) | 1.21 (1.03-1.42) | 1.16 (0.97-1.39) |
| 0–3 years | 1,408 | 55,914 | 292 | 5,548 | 2.09 (1.85-2.37) | 1.21 (1.05-1.39) | 1.17 (1.00-1.36) |
| 0–4 years | 1,658 | 69,916 | 329 | 7,003 | 1.99 (1.77-2.24) | 1.19 (1.04-1.36) | 1.17 (1.01-1.35) |
| 0–5 years | 1,847 | 82,010 | 366 | 8,296 | 1.97 (1.76-2.21) | 1.20 (1.06-1.35) | 1.17 (1.02-1.34) |

Abbreviations: HR, hazard ratio; CI, confidence interval

^a^Adjusted for Charlson Comorbidity Index score.

^b^Complete-case analysis was used to handle missing data in the fully adjusted model. Notably, the number of events, person-years at risk and rate per 1,000 person-years in the bereaved and matched comparators cohorts presented in this table were calculated for the full cohort in the unadjusted and adjusted models only. Adjusted additionally for education duration. In Denmark the total number of patients was 23,853 after excluding those with missing values for education duration.
